# The burden of childhood and adolescent cancers in North Africa and the Middle East (NAME) region: findings from the Global Burden of Disease study 2019

**DOI:** 10.1186/s12887-023-03931-4

**Published:** 2023-03-08

**Authors:** Amirali Karimi, Sahar Saeedi Moghaddam, Sina Azadnajafabad, Zahra Esfahani, Yeganeh Sharifnejad Tehrani, Mohsen Abbasi-Kangevari, Parnian Shobeiri, Seyyed-Hadi Ghamari, Masoud Masinaei, Nazila Rezaei, Sarvenaz Shahin, Elham Rayzan, Negar Rezaei, Bagher Larijani, Farzad Kompani

**Affiliations:** 1 Non-Communicable Diseases Research Center, Endocrinology and Metabolism Population Sciences Institute, Tehran University of Medical Sciences, Tehran, Iran; 2 Kiel Institute for the World Economy, Kiel, Germany; 3 Department of Biostatistics, University of Social Welfare and Rehabilitation Sciences, Tehran, Iran; 4 Department of Epidemiology and Biostatistics, Tehran University of Medical Sciences, Tehran, Iran; 5 Department of Pediatric Hematology and Oncology, Boston Children’s Hospital, Harvard Medical School, Boston, MA USA; 6 Endocrinology and Metabolism Research Center, Endocrinology and Metabolism Clinical Sciences Institute, Tehran University of Medical Sciences, Tehran, Iran; 7 Division of Hematology and Oncology, Children’s Medical Center, Pediatrics Center of Excellence, Tehran University of Medical Sciences, Children’s Medical Center, 62 Qarib St, Keshavarz Blvd, Tehran, 1419733151 Iran

**Keywords:** Childhood and adolescent cancers, Disability-adjusted life years, Global burden of disease, Incidence, mortality, North Africa and Middle East

## Abstract

**Introduction:**

Despite the significant burden of childhood and adolescent cancers, no specific studies recently discussed the burden of cancer in this group in the North Africa and the Middle East (NAME) region. Therefore, we aimed to study the burden of cancers in this group in this region.

**Materials and methods:**

We retrieved the Global Burden of Disease (GBD) data for children and adolescent cancers (0–19 years old) in the NAME region from 1990 to 2019. 21 types of neoplasms were grouped as “neoplasms”, comprising 19 specific cancer groups as well as “other malignant neoplasms” and “other neoplasms”. Three significant parameters of incidence, deaths, and Disability-Adjusted Life Years (DALYs) were studied. The data are presented with 95% uncertainty intervals (UI), and reported rates per 100,000.

**Results:**

In 2019, almost 6 million (95% UI: 4.166 M–8.405 M) new cases and 11,560(9770-13,578) deaths due to neoplasms occurred in the NAME region. Incidence was higher in females (3.4 M), while deaths (6226 of overall 11,560) and DALYs (501,118 of overall 933,885) were estimated as higher in males. Incidence rates did not significantly change since 1990, while deaths and DALYs rates declined significantly. After excluding “other malignant neoplasms” and “other neoplasms”, leukemia was responsible for the highest number of incidence and deaths (incidence: 10,629(8237-13,081), deaths: 4053(3135-5013), followed by brain and central nervous system cancers (incidence: 5897(4192-7134), deaths: 2446(1761-2960)), and non-Hodgkin lymphoma (incidence: 2741 (2237-3392), deaths: 790(645–962)). Incidence rates of neoplasms were similar for most countries, but countries varied more in terms of death rates. Afghanistan 8.9(6.5–11.9), Sudan 6.4(4.5–8.6), and the Syrian Arab Republic 5.6(4.3–8.3) had the highest overall death rates.

**Conclusion:**

The NAME region is observing relatively constant incidence rates and a decreasing pattern in the deaths and DALYs. Despite this success, several countries are lagging behind in development. Different issues such as economic problems, armed conflicts and political instabilities, lack of equipment or experienced staff or poor distribution, stigmatization and disbelief in the healthcare systems account for unfavorable numbers in some countries. Such problems require urgent solutions as new sophisticated and personalized cares raise the alarm for even more inequalities between high and low-income countries.

**Supplementary Information:**

The online version contains supplementary material available at 10.1186/s12887-023-03931-4.

## Introduction

Childhood and adolescent cancers rank as the sixth-highest burdensome cancer group in terms of worldwide disability-adjusted life years (DALYs) [1]. The 5-year survival rates for many of these cancers have improved during recent years in higher-income countries, while the lower socio-demographic index (SDI) quintiles are lagging behind [2]. To make matters worse in these countries, low-to-middle SDI countries share more than 80% of the worldwide DALYs, and childhood and adolescent cancers impose the highest DALYs among various cancer groups in low and low-middle SDI countries [1].

North Africa and the Middle East (NAME) region comprise 21 countries and a considerable portion of them are categorized into low-to-middle income countries, thus carrying a considerable burden of pediatric and adolescent cancers worldwide, as mentioned above [3]. An analysis of the global quality of care for leukemia, an important childhood cancer, demonstrated that most countries in the NAME region had lower than average quality of care [4]. To make matters even worse, the global quality of care index for leukemia had grown in the past 28 years, while the numbers illustrated a steady pattern for the quality of care in the low to low-middle income countries [4]. Many of these countries faced crippling social, economic, and political issues that challenged their healthcare systems, including adequate care for childhood and adolescent cancers [5]. Although the need for calculating the burden of childhood and adolescent cancers in the NAME region is felt deeply, no studies specifically analyzed this matter. The Global Burden of Disease (GBD) study provides a unique opportunity to estimate the burden of hundreds of diseases and risk factors for various regions, including NAME [6]. While we found little to no studies utilizing holistic estimates to describe the important matter of childhood and adolescents’ cancers in the NAME region, GBD 2019 estimates with their powerful modeling and analyzes provide valuable data on this issue. Therefore, we aimed to study and present the burden of childhood and adolescent cancers in the NAME region using the GBD 2019 estimates.

## Materials and methods

### Overview and data sources

We retrieved the data for children and adolescent cancers in the NAME region from 1990 to 2019, made available from IHME (Institute for Health Metrics and Evaluation) “GBD compare” tool available online at https://vizhub.healthdata.org/gbd-compare/ [7], and the GBD 2019 codes can be accessed from https://ghdx.healthdata.org/gbd-2019/code. GBD study 2019 entails the data for 369 injuries and diseases, 286 causes of death, and 87 risk factors in 204 countries and territories [6]. The data input for GBD 2019 comes from vital registration systems, cancer registries, and verbal autopsy reports [8]. The cancer classifications used are derived from the International Statistical Classification of Diseases and Related Health Problems, ninth and tenth revisions (ICD-9 and ICD-10, respectively) [8–10]. GBD 2019 further improved on their data and estimations related to cancer burden from their previous version in 2017 by several means, including using novel modeling and estimation methods and incorporating new data, locations, and years [8]. Therefore, GBD 2019 seemed suitable for the goals of this study.

### NAME region and estimates

NAME is one of the seven GBD super-regions as well as one of the 21 GBD regions and contain 21 countries of Afghanistan, Algeria, Bahrain, Egypt, Iran (the Islamic Republic of), Iraq, Jordan, Kuwait, Lebanon, Libya, Morocco, Oman, Palestine, Qatar, Saudi Arabia, Sudan, Syrian Arab Republic, Tunisia, Turkey, United Arab Emirates, and Yemen. To address the differences in the countries and elicit the current status for the huge number of children and adolescents living in the NAME region, we reported the estimates presented by the GBD 2019 study to provide insights on this issue in the region.

We used three principal measures of incidence, deaths, and DALYs. Incidence of pediatrics and adolescent neoplasms reports the occurrence of new neoplasm cases in a certain period of time (in our study, each designated year). Deaths reports the number of mortalities in that year. DALYs is a time-based measure that combines years of life lost (YLLs) and years lived with disability (YLDs) [11]. Further details on the methods for GBD estimations are previously described [6, 8].

### Cancers and age groups

The data were extracted for 21 countries of the NAME region and 21 neoplasm groups (see Supplementary Table 1 for details on each variable). These 21 neoplasm groups comprised 19 specific malignant neoplasm types, as well as “other malignant neoplasms” and “other neoplasms”. “Other neoplasms” included myelodysplastic, myeloproliferative, and other hematopoietic neoplasms, benign and in situ intestinal neoplasms, benign and in situ cervical and uterine neoplasms, and other benign and in situ neoplasms. GBD code for each neoplasm is also mentioned in Supplementary Table 1.

To better understand the countries and their locations in order to better comprehend the figures that are presented as maps, please refer to Supplementary Fig. 1 that depicts a raw map of the NAME region.

Children and adolescents were defined as 0 to 19 years old. The utilized age subgroups based on the GBD data were: early neonatal (0–6 days), late neonatal (7–27 days), post-neonatal (28–364 days), 1–4 years, 5–9 years, 10–14 years, and 15–19 years.

### Reporting standards

Rates were reported per 100,000 person-years. The data are presented with 95% uncertainty intervals (UI), and UI is used to calculate the level of statistical significance. Calculating 95% UI involved taking 1000 draws for each computational process. The 25th and 975th values represent the UI. All illustrations were carried out using R statistical packages v4.0.4 (http://www.r-project.org/, RRID: SCR_001905).

## Results

### Overview

In 2019, almost 6 million (95% UI: 4.166 M–8.405 M) neoplasms occurred in the NAME region in children and adolescents (0–19 years old), with female patients responsible for most of the cases with an incidence of around 3.4 million (2.4 M–4.8 M). Overall, 933,885 (787,119-1,100,065) DALYs and 11,560 (9770-13,578) deaths were estimated in 2019. DALYs and death were more emphasized in males. DALYs was 501,118 (411,613-602,264) in males and 432,767 (363,008-503,128) in females, and deaths were 6226 (5133-7499) and 5334 (4503-6181), respectively. Incidence rates of neoplasms did not significantly change since 1990 overall in both sexes (incidence rate change: 1.2% (− 0.7 to 3.3%)), but DALYs and deaths significantly decreased in all the groups (DALYs for both sexes: − 36.9% (− 52.7% to − 10.2%), deaths: − 35.9% (− 51.7% to − 9.8%)) (Table 1).

**Table 1:** Overall incidence, DALYs, and mortality of pediatric cancers overall with percent change from 1990 to 2019 and 95% uncertainty intervals (UI)

Location	Sex	Cause	Incidence			DALYs			Deaths		
			Number	Rate per 100,00	Change in rates (%)	Number	Rate per 100,00	Change in rates (%)	Number	Rate per 100,00	Change in rates (%)
North Africa and Middle East	Both	Neoplasms	5,999,741 (4,166,331 to 8,405,857)	2622.3 (1821 to 3674)	1.2 (−0.7 to 3.3)	933,885 (787,119 to 1,100,065)	408.2 (344 to 480.8)	−36.9 (−52.7 to −10.2)	11,560 (9770 to 13,578)	5.1 (4.3 to 5.9)	−35.9 (−51.7 to −9.8)
		Other malignant neoplasms	10,692 (9257 to 12,297)	4.7 (4 to 5.4)	37.2 (8.3 to 69)	230,419 (196,583 to 270,277)	100.7 (85.9 to 118.1)	−18.5 (−39.6 to 4.1)	2848 (2436 to 3351)	1.2 (1.1 to 1.5)	−17.8 (−38.6 to 4.3)
		Other neoplasms	5,961,787 (4,130,600 to 8,366,937)	2605.7 (1805.4 to 3656.9)	1.3 (− 0.6 to 3.5)	3314 (2732 to 3977)	1.4 (1.2 to 1.7)	−35.2 (−52.3 to − 11.3)	33 (27 to 39)	0 (0 to 0)	−37.9 (− 55.9 to −9.6)
	Female	Neoplasms	3,388,895 (2,352,496 to 4,817,646)	3055.3 (2120.9 to 4343.5)	1.2 (−0.8 to 3.4)	432,767 (363,008 to 503,128)	390.2 (327.3 to 453.6)	−37.5 (−53.8 to −9.1)	5334 (4503 to 6181)	4.8 (4.1 to 5.6)	−36.4 (− 52.7 to − 9)
		Other malignant neoplasms	6951 (5888 to 8208)	6.3 (5.3 to 7.4)	54.4 (10.6 to 100)	120,838 (102,027 to 144,788)	108.9 (92 to 130.5)	−15 (−41.5 to 14.9)	1478 (1248 to 1770)	1.3 (1.1 to 1.6)	− 14.9 (− 40.7 to 14.2)
		Other neoplasms	3,369,312 (2,330,301 to 4,797,852)	3037.7 (2100.9 to 4325.6)	1.3 (−0.7 to 3.6)	1671 (1358 to 2027)	1.5 (1.2 to 1.8)	−34.9 (− 63.6 to − 4)	16 (13 to 20)	0 (0 to 0)	−37.4 (− 67.3 to 0)
	Male	Neoplasms	2,610,846 (1,783,507 to 3,674,539)	2214.9 (1513 to 3117.2)	1.5 (−0.8 to 4)	501,118 (411,613 to 602,264)	425.1 (349.2 to 510.9)	−36.3 (− 54.8 to −5)	6226 (5133 to 7499)	5.3 (4.4 to 6.4)	−35.5 (− 53.9 to − 5)
		Other malignant neoplasms	3742 (2977 to 4686)	3.2 (2.5 to 4)	14 (− 21.4 to 58.1)	109,580 (85,829 to 135,157)	93 (72.8 to 114.7)	−22 (− 49.2 to 11)	1370 (1077 to 1692)	1.2 (0.9 to 1.4)	−20.7 (− 47.8 to 11.4)
		Other neoplasms	2,592,474 (1,765,866 to 3,654,106)	2199.3 (1498 to 3099.9)	1.5 (− 0.7 to 4)	1643 (1216 to 2058)	1.4 (1 to 1.7)	− 35.5 (− 58.2 to 2.5)	16 (12 to 21)	0 (0 to 0)	−38.3 (− 61.4 to 6)

Data in parentheses are 95% Uncertainty Intervals (95% UIs); *DALYs* Disability-Adjusted Life Years

“Other neoplasms” (non-malignant) were responsible for 5.962 million (4.131 M–8.367 M) new cases in 2019, 3.369 million (2.330 M–4.798 M) of them in females. Other neoplasms caused few DALYs and deaths (overall DALYs: 3314 (2732-3977), overall deaths: 33 (27–39)) that were not different between females (DALYs: 1671 (1358-2027), deaths: 16 (13-20)) and males (DALYs: 1643 (1216-2058), deaths: 16 (12-21)).

### Cancer-wise

**Fig. 1:**
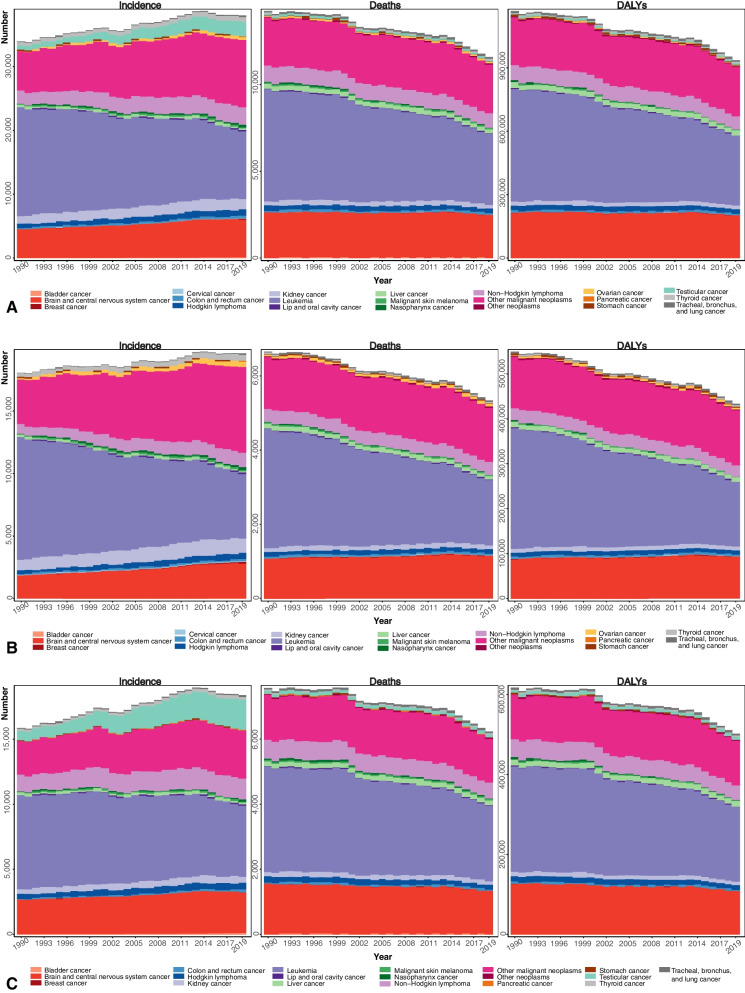
Number of incidences, deaths, and DALYs of pediatric cancers in the NAME region by cancer type for **A**) Overall, **B**) Females, **C**) Males. Note that “other neoplasms” were removed from the “incidence” plots to better visualize other values, as the very high proportion of “other neoplasms” incidence masked the incidence of other cancers

Excluding “other neoplasms” group, “other malignant neoplasms” had the highest incidence in 2019, with 10,692 (9257-12,297) new cases. Besides these two and among specific causes, leukemia, brain and central nervous system (CNS) cancers, and non-Hodgkin lymphoma had the highest incidence in 2019 with 10,629 (8237-13,081), 5897 (4192-7134), and 2741 (2237-3392) cases, respectively. “Other neoplasms” had considerably higher incidence compared to other groups. Figure 1 depicts stacked incidence, deaths, and DALYs for both sexes, and excludes “other neoplasms” from the figure for incidence to better visualize cancer types.

Pancreatic, cervical, and lip and oral cavity cancers had the lowest incidence with 21 (17–28), 63 (47–85), and 97 (83–112), respectively, and all are scoring an incidence rate of below 0.05 per 100,000. Incidence rates of some cancers increased: lip and oral cavity cancer (29.7% change from 1990 to 2019, incidence in 2019: 97), pancreatic cancer (55.2%, 21), malignant skin melanoma (56.8%, 173), breast cancer (118.2%, 161), bladder cancer (120.7%, 161), thyroid cancer (45.9%, 688), Hodgkin lymphoma (38.5%, 1007), and “other malignant neoplasms” (37.2%, 10,692). Other types of cancers did not demonstrate significant changes in incidence rates.

“Other malignant neoplasms” were the second highest cause of death after leukemia, with 2848 (2436-3351) deaths in 2019. Among the specific causes, the highest number of deaths in 2019 was due to leukemia, brain and CNS cancers, and non-Hodgkin lymphoma with 4053 (3135-5013), 2446 (1761-2960), and 790 (645–962) deaths. The least mortalities were related to cervical (10 (7-13)), pancreatic (13 (10-17)), and bladder cancers (18 (10–34)). Despite the very high incidence rates, “other neoplasms” caused few deaths. Death rates of most cancers showed a declining trend since 1990, but nasopharynx cancer (− 50% change from 1990 to 2019, deaths in 2019: 60), stomach cancer (− 36.1%, 55), thyroid cancer (− 31.3%, 32), Hodgkin lymphoma (− 35.5%, 225), and leukemia (− 50.2%, 4053) reached statistical significance, and the death rate for pancreatic cancer (49.0%, 13) increased in this period. DALYs also mostly declined during the period (Table 2).

**Table 2:** Incidence, DALY, and mortality of various cancers in the North Africa and middle-East region in 2019, with 95% uncertainty intervals (UI)

Cancer type	Incidence			DALYs			Deaths		
	Number	Rate per 100,000	% change in rate from 1990 to 2019	Number	Rate per 100,000	% change in rate from 1990 to 2019	Number	Rate per 100,000	% change in rate from 1990 to 2019
Lip and oral cavity cancer	97 (83 to 112)	0.04 (0.04 to 0.05)	29.74 (8.43 to 54.36)	1893 (1618 to 2189)	0.83 (0.71 to 0.96)	1.57 (−14.97 to 21.02)	24 (21 to 28)	0.01 (0.01 to 0.01)	1.30 (−15.11 to 21.21)
Nasopharynx cancer	276 (234 to 326)	0.12 (0.10 to 0.14)	8.71 (−14.95 to 42.27)	4622 (3856 to 5545)	2.02 (1.69 to 2.42)	−49.35 (−60.81 to − 31.58)	60 (50 to 72)	0.03 (0.02 to 0.03)	− 49.96 (− 61.43 to − 32.29)
Stomach cancer	100 (83 to 122)	0.04 (0.04 to 0.05)	−29.33 (−44.20 to − 11.40)	3997 (3249 to 4920)	1.75 (1.42 to 2.15)	−36.12 (− 50.11 to − 17.95)	55 (45 to 68)	0.02 (0.02 to 0.03)	− 36.14 (− 50.13 to − 17.96)
Colon and rectum cancer	262 (220 to 316)	0.11 (0.10 to 0.14)	13.01 (−19.35 to 58.44)	7915 (6639 to 9484)	3.46 (2.90 to 4.14)	−18.44 (− 42.47 to 16.43)	104 (87 to 124)	0.05 (0.04 to 0.05)	−17.61 (−41.73 to 17.21)
Liver cancer	359 (271 to 472)	0.16 (0.12 to 0.21)	−15.95 (−39.77 to 17.30)	22,560 (17,312 to 30,839)	9.86 (7.57 to 13.48)	−33.84 (−56.89 to 0.98)	280 (218 to 378)	0.12 (0.10 to 0.17)	−31.99 (−54.82 to 1.50)
Pancreatic cancer	21 (17 to 28)	0.01 (0.01 to 0.01)	55.22 (14.67 to 113.41)	913 (712 to 1211)	0.40 (0.31 to 0.53)	48.89 (10.03 to 103.55)	13 (10 to 17)	0.01 (0 to 0.01)	48.97 (10.12 to 103.68)
Tracheal, bronchus, and lung cancer	160 (135 to 191)	0.07 (0.06 to 0.08)	4.29 (−20.57 to 47.64)	7584 (6403 to 9071)	3.31 (2.80 to 3.96)	−1.28 (−25.84 to 40.15)	104 (87 to 124)	0.05 (0.04 to 0.05)	−0.80 (− 25.30 to 40.58)
Malignant skin melanoma	173 (143 to 221)	0.08 (0.06 to 0.10)	56.85 (3.15 to 136.25)	2496 (2074 to 3121)	1.09 (0.91 to 1.36)	−27.59 (−53.38 to 7.32)	31 (26 to 39)	0.01 (0.01 to 0.02)	−28.16 (−52.87 to 4.68)
Breast cancer	161 (129 to 205)	0.07 (0.06 to 0.09)	118.20 (58.20 to 197.56)	2231 (1703 to 2975)	0.98 (0.74 to 1.30)	36.20 (−4.15 to 98.12)	30 (23 to 40)	0.01 (0.01 to 0.02)	33.69 (−5.78 to 95.13)
Cervical cancer	63 (47 to 85)	0.03 (0.02 to 0.04)	−6.07 (−31.24 to 37.41)	715 (526 to 984)	0.31 (0.23 to 0.43)	−29.99 (−49.20 to 1.36)	10 (7 to 13)	0 (0 to 0.01)	−30.90 (− 49.88 to 0.75)
Ovarian cancer	413 (293 to 528)	0.18 (0.13 to 0.23)	96.41 (−18.64 to 216.25)	5645 (4065 to 7196)	2.47 (1.78 to 3.15)	40.27 (−39.43 to 125.71)	72 (52 to 92)	0.03 (0.02 to 0.04)	39.82 (−39.45 to 124.66)
Testicular cancer	2325 (1383 to 3886)	1.02 (0.6 to 1.70)	133.53 (−10.98 to 454.38)	7967 (6264 to 10,091)	3.48 (2.74 to 4.41)	−21.33 (−59.16 to 38.60)	84 (67 to 104)	0.04 (0.03 to 0.05)	−29.08 (−63.25 to 22.50)
Kidney cancer	1728 (1336 to 2124)	0.76 (0.58 to 0.93)	3.18 (−41.08 to 48.99)	21,446 (16,921 to 26,207)	9.37 (7.40 to 11.45)	−28.33 (−56.28 to 6.48)	248 (196 to 302)	0.11 (0.09 to 0.13)	−28.27 (−55.89 to 6.13)
Bladder cancer	161 (95 to 313)	0.07 (0.04 to 0.14)	120.71 (15.21 to 319.44)	1380 (798 to 2638)	0.60 (0.35 to 1.15)	22.99 (−37.42 to 138.86)	18 (10 to 34)	0.01 (0 to 0.01)	18.11 (−39.47 to 127.46)
Brain and central nervous system cancer	5897 (4192 to 7134)	2.58 (1.83 to 3.12)	6.02 (−44.54 to 75.38)	198,428 (142,686 to 240,395)	86.73 (62.36 to 105.07)	−26.80 (−59.93 to 21.09)	2446 (1761 to 2960)	1.07 (0.77 to 1.29)	−25.88 (−59.12 to 21.31)
Thyroid cancer	688 (481 to 874)	0.30 (0.21 to 0.38)	45.93 (2.83 to 105.37)	2825 (2005 to 3577)	1.23 (0.88 to 1.56)	−27.73 (−49.38 to 2.88)	32 (23 to 41)	0.01 (0.01 to 0.02)	−31.30 (−51.46 to − 3.25)
Hodgkin lymphoma	1007 (713 to 1240)	0.44 (0.31 to 0.54)	38.54 (9.70 to 72.28)	17,512 (12,799 to 22,132)	7.65 (5.59 to 9.67)	−34.67 (−47.89 to −16.43)	225 (165 to 286)	0.1 (0.07 to 0.12)	−35.48 (−48.49 to −17.41)
Non-Hodgkin lymphoma	2741 (2237 to 3392)	1.20 (0.98 to 1.48)	11.77 (−22.24 to 76.56)	62,451 (50,936 to 76,027)	27.30 (22.26 to 33.23)	−33.76 (−55.82 to 14.12)	790 (645 to 962)	0.35 (0.28 to 0.42)	−33.23 (−55.17 to 13.56)
Leukemia	10,629 (8237 to 13,081)	4.65 (3.60 to 5.72)	−50.53 (−68.66 to −12.36)	327,572 (252,032 to 406,381)	143.17 (110.16 to 177.62)	−51.48 (−66.78 to −22.55)	4053 (3135 to 5013)	1.77 (1.37 to 2.19)	−50.25 (−65.59 to −22.00)
Other malignant neoplasms	10,692 (9257 to 12,297)	4.67 (4.05 to 5.37)	37.24 (8.34 to 68.99)	230,419 (196,583 to 270,277)	100.71 (85.92 to 118.13)	−18.49 (−39.64 to 4.13)	2848 (2436 to 3351)	1.24 (1.06 to 1.46)	−17.80 (−38.64 to 4.27)
Other neoplasms	5,961,787 (4,130,600 to 8,366,937)	2605.72 (1805.37 to 3656.95)	1.33 (−0.56 to 3.47)	3314 (2732 to 3977)	1.45 (1.19 to 1.74)	−35.20 (−52.26 to −11.35)	33 (27 to 39)	0.01 (0.01 to 0.02)	−37.85 (−55.89 to −9.61)

Data in parentheses are 95% Uncertainty Intervals (95% UIs); *DALYs* Disability-Adjusted Life Years

**Fig. 2:**
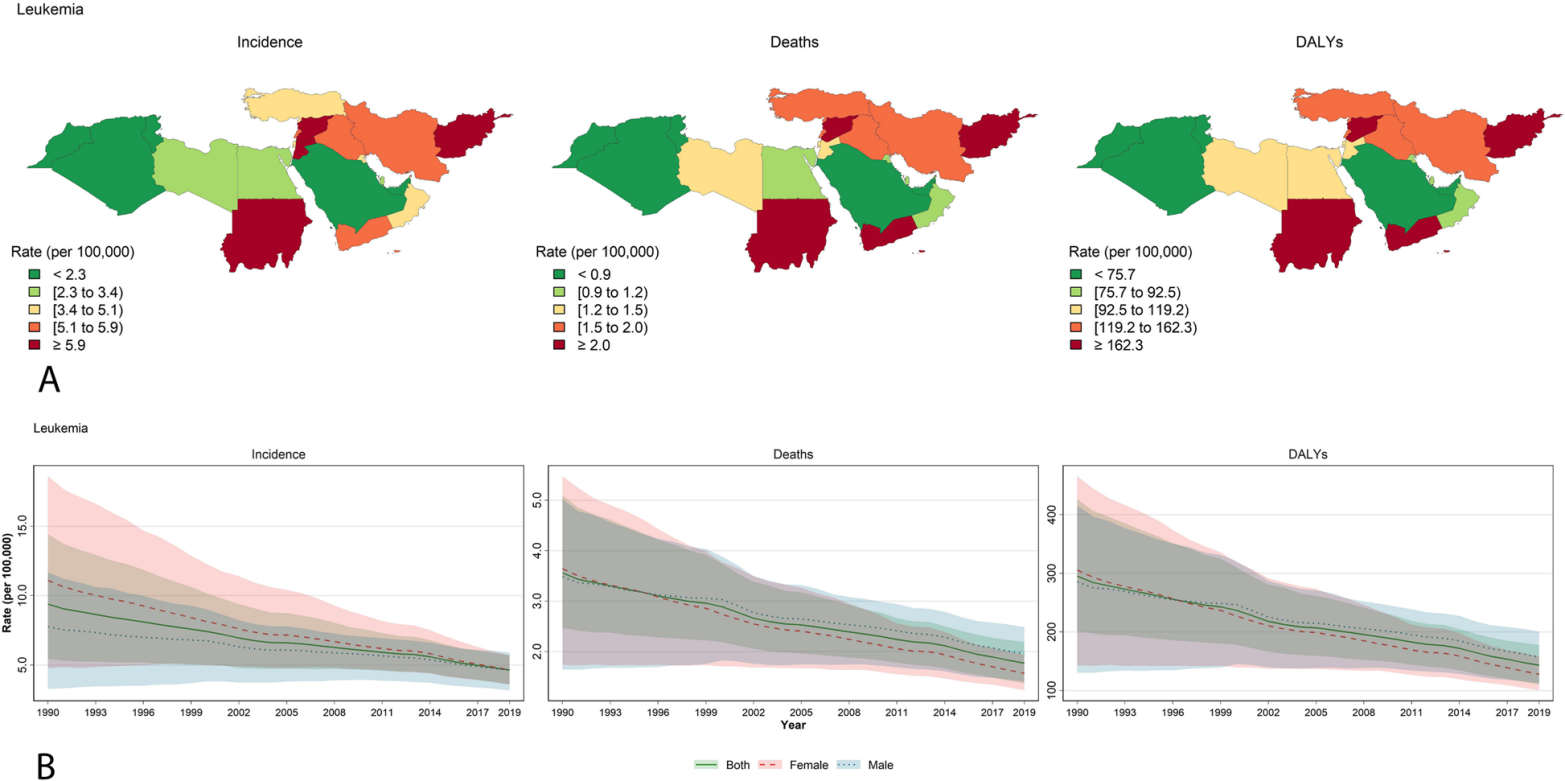
Details for leukemia in the NAME region: **A** Geographical distribution of the incidence, deaths, and DALYs rate (per 100,000 population) by the countries of the region in 2019 for both sex; **B** Trends for incidence, deaths, and DALYs rate (per 100,000 population) from 1990 to 2019 for both sexes, males, and females. The lines present the values for both sexes, males, and females, and the shadows around each line with the same color present the uncertainty intervals (UI) for that line. Maps are republished from https://www.openstreetmap.org/ under a CC BY license, with permission from https://www.openstreetmap.org/copyright, original copyright 2020)

**Fig. 3:**
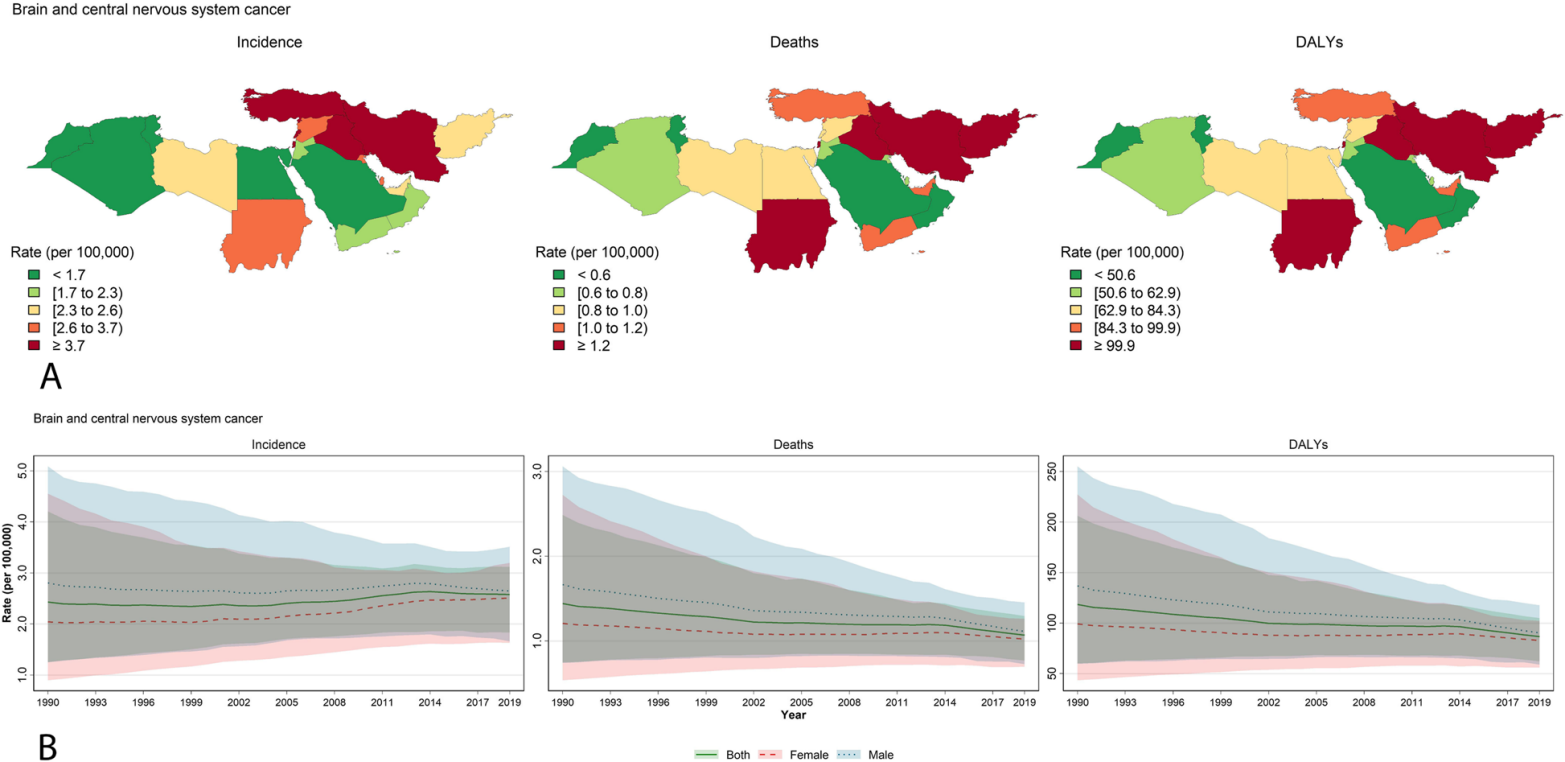
Details for brain and CNS cancers in the NAME region: **A** Geographical distribution of the incidence, deaths, and DALYs rate (per 100,000 population) by the countries of the region in 2019 for both sex; **B** Trends for incidence, deaths, and DALYs rate (per 100,000 population) from 1990 to 2019 for both sexes, males, and females. The lines present the values for both sexes, males, and females, and the shadows around each line with the same color present the uncertainty intervals (UI) for that line. Maps are republished from https://www.openstreetmap.org/ under a CC BY license, with permission from https://www.openstreetmap.org/copyright, original copyright 2020)

**Fig. 4:**
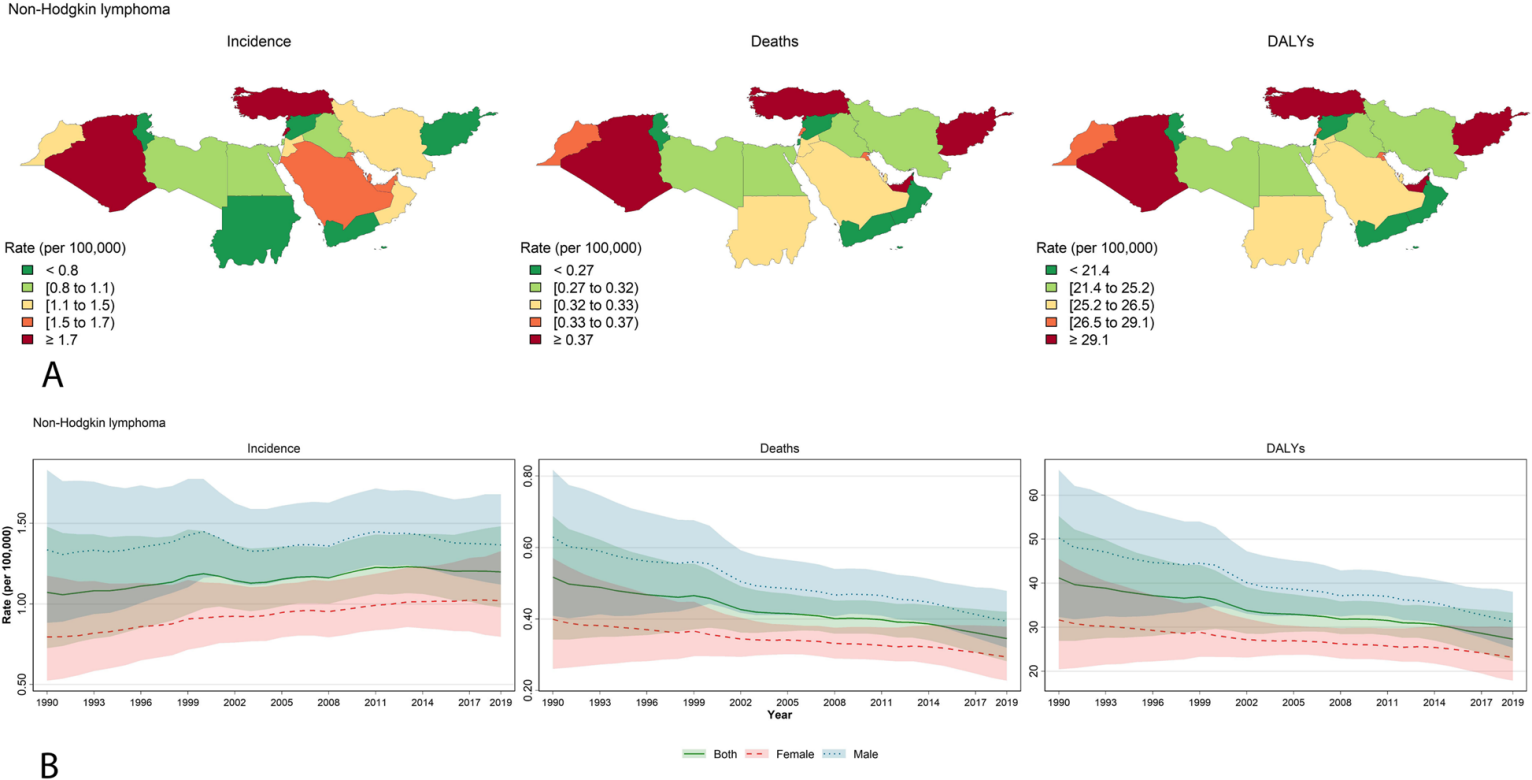
Details for non-Hodgkin lymphoma in the NAME region: **A** Geographical distribution of the incidence, deaths, and DALYs rate (per 100,000 population) by the countries of the region in 2019 for both sex; **B** Trends for incidence, deaths, and DALYs rate (per 100,000 population) from 1990 to 2019 for both sexes, males, and females Maps are republished from https://www.openstreetmap.org/ under a CC BY license, with permission from https://www.openstreetmap.org/copyright, original copyright 2020)

Figures 2, 3 and 4 were plotted explicitly for leukemia, brain and CNS cancers, and non-Hodgkin lymphoma which had the most incidence and deaths among the specific causes, and are considered the most critical childhood and adolescent cancers [1]. Figures 2A, 3A, and 4A illustrate the maps for incidence, deaths, and DALYs based on the countries. Leukemia demonstrates a steady decrease in all three parameters of incidence, deaths, and DALYs (Fig. 2B). Despite the slightly increasing trend in the incidence of brain and CNS cancers, deaths and DALYs were decreasing (Fig. 3B). Similar to brain and CNS cancers, non-Hodgkin lymphoma also increased incidence but decreased DALY and deaths from 1990 to 2019 (Fig. 4B). Incidence, deaths, and DALYs rates of leukemia and brain and CNS cancers are the highest in the early neonatal period, then decline until reaching their lowest values in the 15–19 age group (Supplementary Figs. 2 and 3). On the other hand, non-Hodgkin lymphoma had the highest incidence, DALY, and prevalence in the 15–19 years old age group (Supplementary Fig. 4). Rankings of the countries are also mentioned in Supplementary Figs. 5, 6 and 7.

### Country-wise

Incidence rates of “total neoplasms” ranged similarly between 2529 and 2747 per 100,000 for most of the countries. Exceptions were Jordan 2047 (1467-2793), Turkey 3098 (2106-4278), and Qatar 3321 (2307-4638). Incidence rates of “total neoplasms” tended to be higher in females than males in all the countries.

Afghanistan 8.9 (6.5–11.9), Sudan 6.4 (4.5–8.6), and the Syrian Arab Republic 5.6 (4.3–8.3) had the highest overall death rates per 100,000 for neoplasms. Most of the countries tended to have more deaths related to “total neoplasms” in males than females, except Afghanistan, Libya, Morocco, Saudi Arabia, and Tunisia where the deaths were higher in females. Afghanistan ranked first in both sexes, but the numbers were higher for females (females: 10.2 (7.3–14.0), males: 7.6 (4.8–10.9)). The lowest death rates for the neoplasms group belonged to Saudi Arabia with 2.3 (1.8–2.8), Tunisia with 2.5 (2.0–3.2), and Morocco with 2.8 (2.2–3.5). All the countries had an acceptable reduction in mortality rates since 1990, ranging from 15.7% in Oman to 51% in Turkey (Supplementary Table 2).

## Discussion

The incidence rates of pediatric cancers remained constant in the NAME region, and DALYs and mortality are decreasing. Female patients had a higher incidence with 3.4 of 6 million in 2019, but suffered fewer deaths and DALYs than males. Excluding “other neoplasms” and “other malignant neoplasms”, Leukemia and brain and CNS cancers were the first and second group of neoplasms in incidence and death rates. Most cancers had declining death and DALYs rates. Incidence rates of neoplasms were similar for most countries, but countries varied more in terms of death rates. Afghanistan, Sudan, and the Syrian Arab Republic had the highest death rates, while Saudi Arabia, Tunisia, and Morocco had the least deaths. All the countries have made progress in lowering their death rates since 1990.

This study’s trends were similar to the latest published global GBD estimate for children and adolescent cancers in 2017. Similar to our study, leukemia, uncategorized cancers, and brain and CNS cancers were the most important causes of incidence and mortality in childhood and adolescents, respectively, followed by non-Hodgkin lymphoma [1]. The GBD 2017 study found a higher proportion of children’s leukemia and brain and CNS cancers, while other rare cancers such as thyroid, testes, and ovaries were most prevalent in 15–19 years old adolescents [1]. Non-Hodgkin lymphoma was among the three most common causes of cancer-related DALY in the middle to low SDI countries, while it was the fifth cause in high and high-middle SDI countries [1]. As most of the countries in the NAME region are middle to SDI countries, this finding of GBD 2017 study concurs with our findings. Concurrently, we also found that the incidence of both leukemia and brain and CNS cancers decreased with an increase in age, and the lowest incidence of leukemia and brain and CNS cancers in our study was also in the 15–19 age group. Such findings indicate similar trends between the global data and the NAME region. Leukemia and brain and CNS cancers were also ranked first and second worldwide childhood cancers in both incidence and mortality in the GLOBACAN 2018 study.

Interestingly, non-Hodgkin lymphoma ranked third in the worldwide incidence in the GLOBACAN 2018, while it even overtook brain and CNS cancers in countries with low human development index (HDI) and ranked second [12]. Both GBD 2017 and GLOBACAN 2018 studies did not present their data for various cancers region-wise to potentiate further comparisons specific to the NAME region. The most important difference between our study and the GLOBOCAN 2018 was that they found higher incidence rates in males for both of the Northern Africa and Western Asia regions compared to females, discordant to our findings.

NAME region consists of many countries with low or middle incomes. Survival rates have increased for various childhood cancers in high-income countries, while many lower-income countries are struggling to reach the desired goal [5]. Furthermore, prevention does not have the same critical role in childhood and adolescent cancers as in adult cancers [13]. Although reductions in mortality were observed in all the countries of the region, several countries, especially some lower income countries such as Afghanistan, still face high mortality rates, a finding that hints the need for deeper efforts. The number of cases will also increase in the coming years with the increase in overall population of the NAME region [1], alarming the benefits of early interventions. All these facts emphasize the importance of offering updated advances in diagnosis and treatment in the children and adolescents, improving the training of their medical doctors and specialists, and enhancing the function of their referral systems to decrease their costs. NAME countries should specifically target earlier diagnosis to improve the outcome of their patients because lower-income countries usually detect their patients at higher stages, and they may observe more substantial advantages of early detection compared to the higher-income countries [14]. The countries should increase public awareness and provide adequate diagnostic methods to achieve the goal of earlier diagnosis, as no screening tests have seemed useful for pediatric and adolescent cancers [14, 15].

Fortunately, all the countries in the NAME region have improved death rates since 1990; however, these decreases differed between the countries, and some countries still face high death rates. The issue is multifactorial and should be looked upon from various viewpoints. Cancer diagnosis and treatment require a high amount of investment, and as with other lower-income countries, several countries in the NAME region struggle to allocate the necessary budget to fight this disease. The economic problems will lead to several adverse outcomes; such as later diagnosis due to inadequate diagnosis equipment, shortage of up-to-date treatments due to their high costs, e.g. immunotherapies, low number of hospital beds, decreased investments in growing the public awareness, and financial instability of the families and their lack of health insurance that will deprive them of the vital access to healthcare services [4, 5, 12, 14]. Hence, countries should prioritize childhood and adolescent cancers, as they were classified as very cost-effective by the World Health Organization (WHO) [16].

Armed conflicts have also aggravated the issues in several countries. In the short term, these wars will break the health and social services, target access to healthcare facilities, and decrease the region’s safety, resulting in inadequate diagnosis and treatment. In the long-term, the armed conflicts that damage a country’s infrastructure may require years to be fixed and may also destabilize a country’s political situation and deprioritize healthcare issues and research for the government [17–19]. Furthermore, wars also bring about massive population displacements and provide a ground for higher transmission of infectious diseases that may be responsible for some forms of cancers [18]. Afghanistan is an example that has the highest death rates. They were forced into several exhausting wars with the Soviet Union, and then they suffered from internal conflicts that are still present [17]. Sudan and the Syrian Arab republic also shared similar fates [20] and are among the countries with the highest mortality rates. Such crippling wars pose issues to the healthcare system, and the countries should try to fix the issues during and after the war. Murray et al. proposed using forecasting models to predict the probability of armed conflicts to prevent wars and the collaboration of political and health experts to limit the advent of wars and their destructive outcomes [18]. Other challenges in the NAME region that should be addressed are the presence of concurrent disease that complicates the diagnosis, stigma, lack of medical expertise or poor distribution of the experts in some areas, or disbelief in the healthcare system [5].

Gender disparities should also be taken into consideration. Males suffered from more mortality to incidence ratio understandable from higher incidence of neoplasms in females but higher deaths in males. However, some countries (i.e. Afghanistan, Libya, Morocco, Saudi Arabia, and Tunisia) had higher death rates in females rather than males. These exceptions of higher deaths can hypothetically, and partly, be attributed to gender disparities in access to healthcare. Gender disparities in healthcare allocation can arise from disparities in available health services and the social determinants of health that are separate from the formal healthcare system and are highly influenced by income and education [21]. However, a study on the quality of care of pediatric and adolescent cancers found a similar quality of care index for all the SDI quintiles worldwide [4]. GLOBOCAN 2018 study found more emphasized male predominance in pediatric cancer incidence in lower-income countries, and the authors attributed this to gender inequities that limit the access to healthcare services for females, resulting in under-diagnosis of their neoplasms [12]. Other factors besides sexual disparities may exist and account for this matter, for example, three of the five countries are the North African countries of Libya, Morocco, and Tunisia, and specific regional causes may play important roles that fall beyond the scope of this study. Nevertheless, we also observed higher mortality to incidence ratio in male patients and should not disregard any possible disparities and cultural issues aimed at males that may increase their susceptibility to more advanced diseases in most of the countries.

The disparities also exist at subnational levels in the region. A study on the childhood cancer burden in Iran found inequalities of more than 40% in some provinces [22]. The lack of specialized centers and pediatric oncologists and the higher cost of antineoplastic treatments in some regions decrease healthcare accessibility, increase health costs for the families, and cause unemployment and migration [23, 24]. The authors also proposed several more reasons for the disparities, such as the inhabitants’ health behaviors, level of education and awareness, urban/rural ratios, and specific genetic and oncologic patterns of cancers [25, 26]. We can hypothesize even harsher subnational inequities for the countries that face social and political problems in specific parts, such as the countries where armed conflicts and terrorist groups tear their specific regions.

Childhood and adolescent cancers impose a significant burden internationally. GBD 2017 study ranked childhood and adolescent cancers as the group with the sixth-highest DALYs worldwide, ranking first in the low and low-middle SDI countries [1]. Due to the importance of this matter, the WHO Global Initiative for Childhood Cancer was formed. This initiative called for implementing capacity-building interventions and incorporating childhood cancers into the national cancer policies [27]. International collaboration is required to decrease the burden and reach the determined goals [28]. Low and middle-income countries should be emphasized in the worldwide programs, as they share the most cases and DALYs worldwide and face more economical, social, and political obstacles.

### Limitations

This study is the first study to map such large-scale data for childhood and adolescent cancers in the NAME region and may provide specific insights for the healthcare policy-makers of this region. Nevertheless, it has some limitations. First and foremost, GBD databases may have inaccuracies in primary data sources and estimations, especially the lower-income countries with less adequate surveillance systems [8]. Such estimations might become even more challenging for less prevalent cancers and those requiring sophisticated diagnostics technologies. Regarding this, GBD 2019 study tried to clarify their references and utilize complex modelling techniques to achieve the most precise estimations [6, 29].

Another limitation is related to the unavailability of specific mapping for different types of neoplasms in children and adolescent. In our study, we observed that many neoplasms fell into the categories of “other neoplasms” or “other malignant neoplasms”, which in fact were among the highest causes of new cases and deaths. The GBD estimates and data can improve with developing specific categorizations for children and adolescent to better summarize their neoplasms in accurate groups. For instance, some important childhood cancers such as neuroblastoma or retinoblastoma are lacking in the GBD 2019 presentation system, and their presence can substantially improve the derived conclusions.

Another limitation comes from the timeline of the GBD 2019 estimates, as lack of data from 2019 onwards deprives us from comprehending the effects of COVID-19 pandemic on cancer incidence and mortality. Furthermore, the aim of the study limited us to include some deeper details, such as the detailed analysis of the causes of the observed patterns of incidence/DALYs/deaths changes in every location-cancer. Such follow-up analyzes would be the subjects of future works.

## Conclusion

The NAME region is observing relatively constant incidence rates and a decreasing pattern in the deaths and DALYs. Despite this success, several countries such as Afghanistan, Sudan, and the Syrian Arab republic suffer from high death rates that require urgent attention. Several reasons halt the advancements in some of the NAME’s countries, especially those with high death rates, including economic issues and the low income of the governments and the households, lack of up-to-date equipment for diagnosis and treatment due to the economic strain, armed conflicts and political instabilities, lack of experienced healthcare staff or their poor distribution, stigmatization, and disbelief in the healthcare system. Such issues require urgent care as if they remain, the developing personalized and sophisticated methods might increase the gaps in the quality of cancer care between the lower-income countries of the region and high-SDI countries. Politicians and healthcare policy-makers should evaluate and identify the causes that halt their country’s development and try to fix these issues using international collaborations and expert panels.

Future studies are required to evaluate the changing patterns in the region. These studies need to address the shortcomings of our study and the datasets should contain a more detailed and specific approach towards pediatric and adolescent cancers; as discussed in the limitations, the GBD 2019 estimates’ neoplasm categories were not specifically designed for this age groups. Data quality should improve and the countries should start well-designed and controlled cancer registries to provide quality evidence to their researchers and international databases, thereby increasing the accuracy of future estimates to better follow their implemented strategies. Programs should account for gender differences in the cancers to provide adequate needs of both sexes, and try to detect and eliminate any existing sex-based discriminations as implied by this study. Progresses of the countries should be acknowledged, for instance, all the countries have demonstrated decreased mortalities in our study. The reasons for such progresses should be reinforced, yet the causes for higher mortality rates in some countries should be studied and closely monitored until the mortality rates decline.

## Supplementary Information

**Additional file 1: Supplementary Fig. 1.** Raw map of the NAME region illustrating its countries and their locations. **Supplementary Fig. 2.** Pyramid of the rates of incidence, deaths, and DALYs of leukemia in the NAME region in 1990 and 2019 for males and females in various age subgroups. **Supplementary Fig. 3.** Pyramid of the rates of incidence, deaths, and DALYs of the brain and CNS cancers in the NAME region in 1990 and 2019 for males and females in various age subgroups. **Supplementary Fig. 4.** Pyramid of the rates of incidence, deaths, and DALYs of non-Hodgkin Lymphoma in the NAME region in 1990 and 2019 for males and females in various age subgroups. Note that the data for children below 1 year old and incidence for 1–4 years old were not mapped due to the lack of data. **Supplementary Fig. 5.** Arrow chart of the positions of the NAME countries in leukemia incidence, deaths, and DALYs from 1990 to 2019. The results are reported as rates per 100,000. **Supplementary Fig. 6.** Arrow chart of the positions of the NAME countries in brain and CNS cancers incidence, deaths, and DALYs from 1990 to 2019. The results are reported as rates per 100,000. **Supplementary Fig. 7.** Arrow chart of the positions of the NAME countries in non-Hodgkin lymphoma incidence, deaths, and DALYs from 1990 to 2019. The results are reported as rates per 100,000. **Supplementary Table 1.** Details of the countries and the studied cancers. **Supplementary Table 2.** The incidence, deaths, and DALYs of pediatric cancers overall by number and rate (per 100,000) with percent change in rate from 1990 to 2019 and 95% uncertainty intervals (UI) in the NAME countries, by sex.

## Data Availability

Data were extracted using the GBD database available publicly at https://vizhub.healthdata.org/gbd-results/. Data are available upon a request to the corresponding author.
