# Supplementary material for: The burden of childhood and adolescent cancers in North Africa and the Middle East (NAME) region: findings from the Global Burden of Disease study 2019
Source: BMC Pediatr. 2023 Mar 8;23:113. doi: 10.1186/s12887-023-03931-4 (PMC9992906; doi:10.1186/s12887-023-03931-4)
Supplement: Supplementary file 1 — Additional file 1: Supplementary Fig. 1. Raw map of the NAME region illustrating its countries and their locations. Supplementary Fig. 2. Pyramid of the rates of incidence, deaths, and DALYs of leukemia in the NAME region in 1990 and 2019 for males and females in various age subgroups. Supplementary Fig. 3. Pyramid of the rates of incidence, deaths, and DALYs of the brain and CNS cancers in the NAME region in 1990 and 2019 for males and females in various age subgroups. Supplementary Fig. 4. Pyramid of the rates of incidence, deaths, and DALYs of non-Hodgkin Lymphoma in the NAME region in 1990 and 2019 for males and females in various age subgroups. Note that the data for children below 1 year old and incidence for 1–4 years old were not mapped due to the lack of data. Supplementary Fig. 5. Arrow chart of the positions of the NAME countries in leukemia incidence, deaths, and DALYs from 1990 to 2019. The results are reported as rates per 100,000. Supplementary Fig. 6. Arrow chart of the positions of the NAME countries in brain and CNS cancers incidence, deaths, and DALYs from 1990 to 2019. The results are reported as rates per 100,000. Supplementary Fig. 7. Arrow chart of the positions of the NAME countries in non-Hodgkin lymphoma incidence, deaths, and DALYs from 1990 to 2019. The results are reported as rates per 100,000. Supplementary Table 1. Details of the countries and the studied cancers. Supplementary Table 2. The incidence, deaths, and DALYs of pediatric cancers overall by number and rate (per 100,000) with percent change in rate from 1990 to 2019 and 95% uncertainty intervals (UI) in the NAME countries, by sex. [file 12887_2023_3931_MOESM1_ESM.docx]

Supplementary material

**For the article “The burden of childhood and adolescent cancers in North Africa and the Middle East (NAME) region: findings from the Global Burden of Disease Study 2019”**

Supplementary Figure 1. Page 2.

Supplementary Figure 2. Page 3.

Supplementary Figure 3. Page 4.

Supplementary Figure 4. Page 5.

Supplementary Figure 5. Page 6.

Supplementary Figure 6. Page 7.

Supplementary Figure 7. Page 8.

Supplementary Table 1. Page 9.

Supplementary Table 2. Page 10.


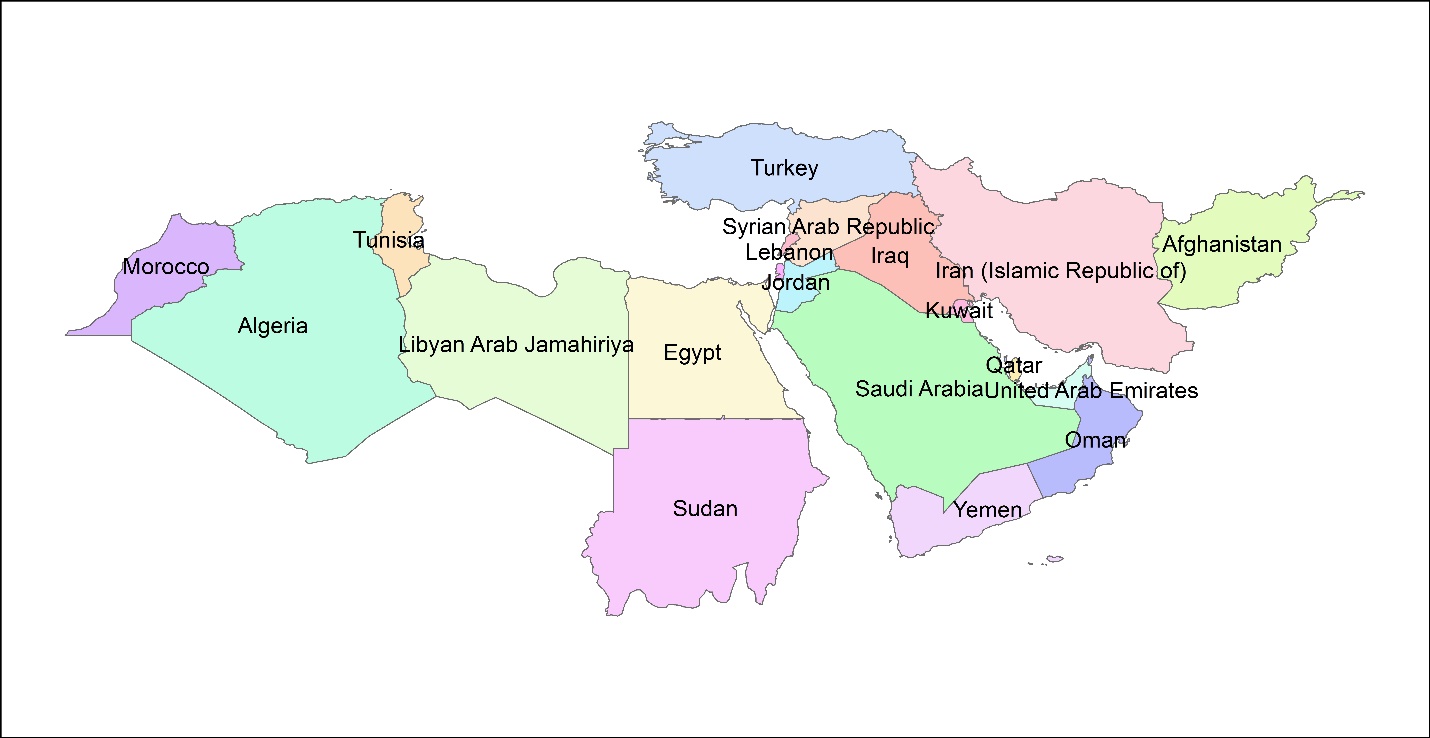


**Supplementary Figure 1.** Raw map of the NAME region illustrating its countries and their locations


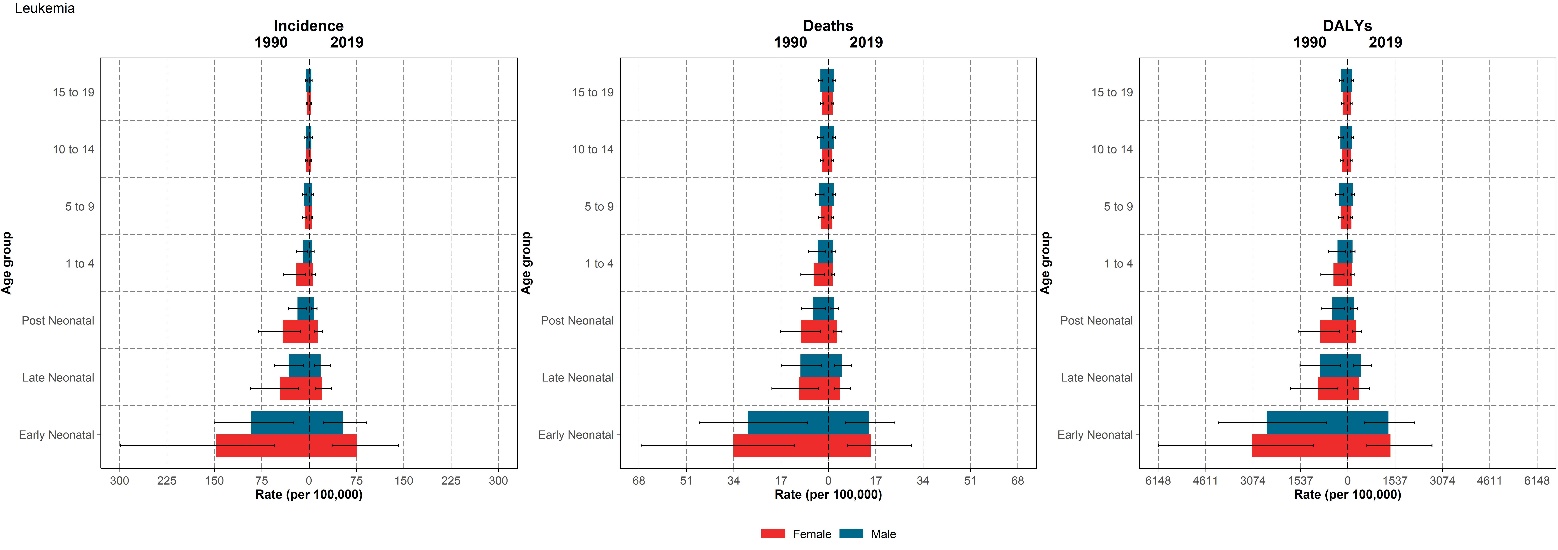


**Supplementary Figure 2:** Pyramid of the rates of incidence, deaths, and DALYs of leukemia in the NAME region in 1990 and 2019 for males and females in various age subgroups


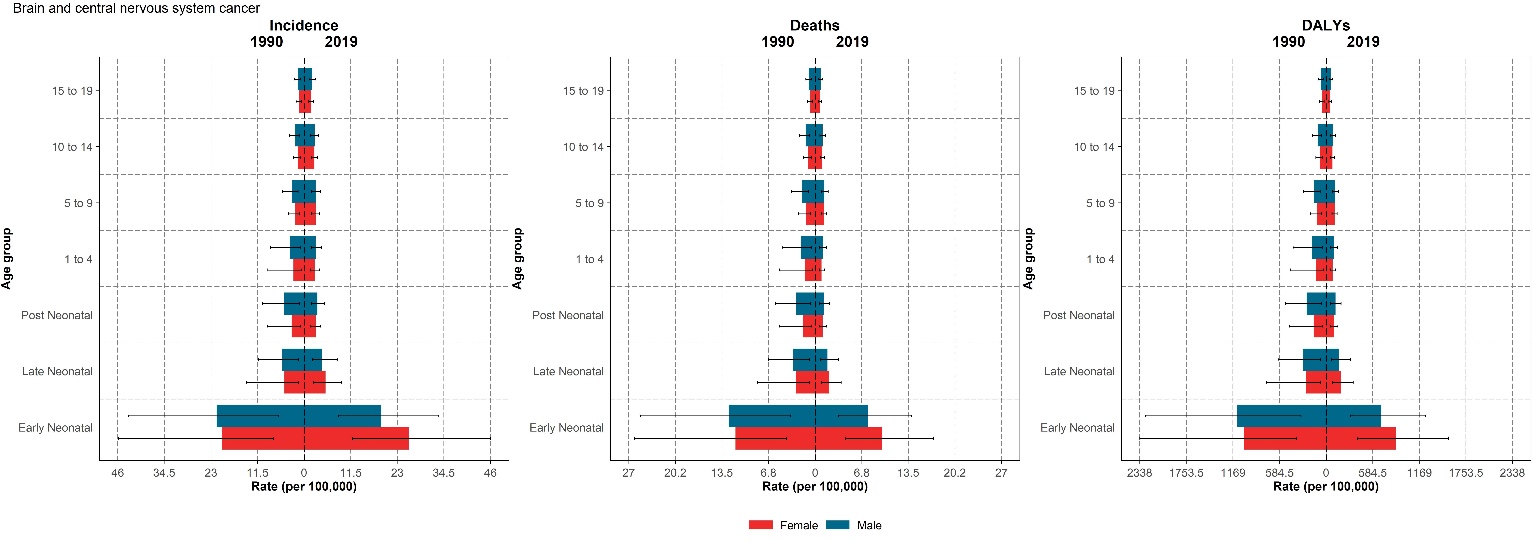


**Supplementary Figure 3:** Pyramid of the rates of incidence, deaths, and DALYs of the brain and CNS cancers in the NAME region in 1990 and 2019 for males and females in various age subgroups


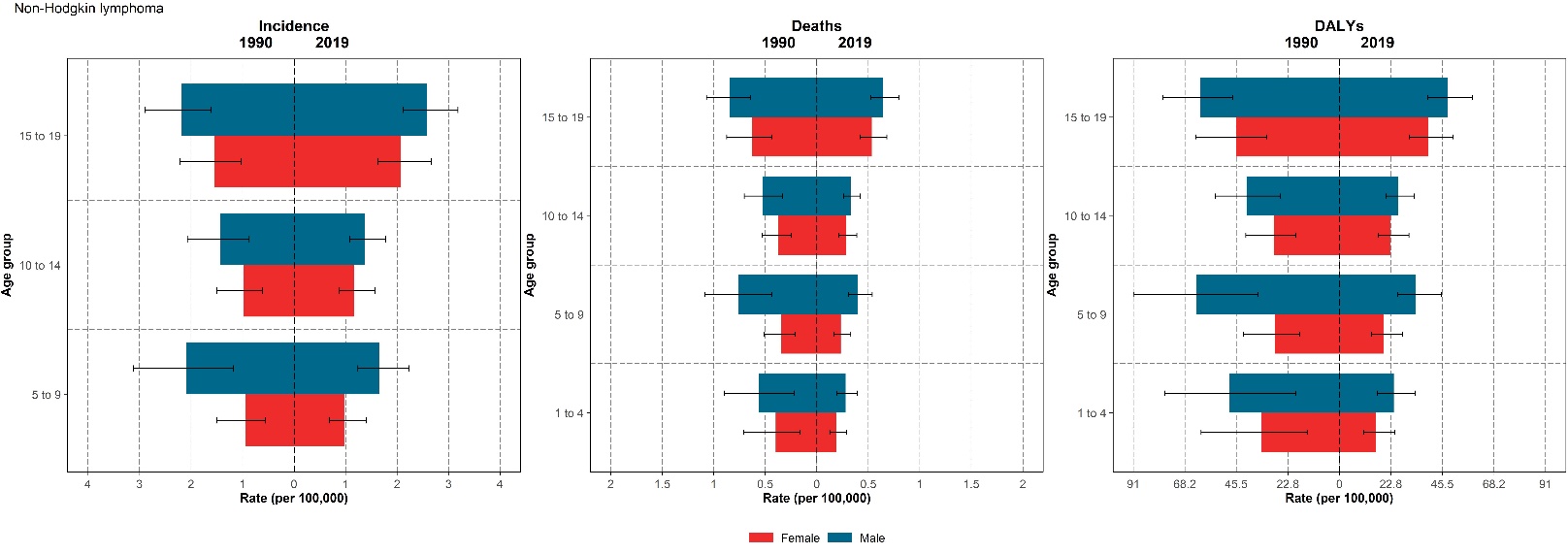
 **Supplementary Figure 4:** Pyramid of the rates of incidence, deaths, and DALYs of non-Hodgkin Lymphoma in the NAME region in 1990 and 2019 for males and females in various age subgroups. Note that the data for children below one year old and incidence for 1-4 years old were not mapped due to the lack of data


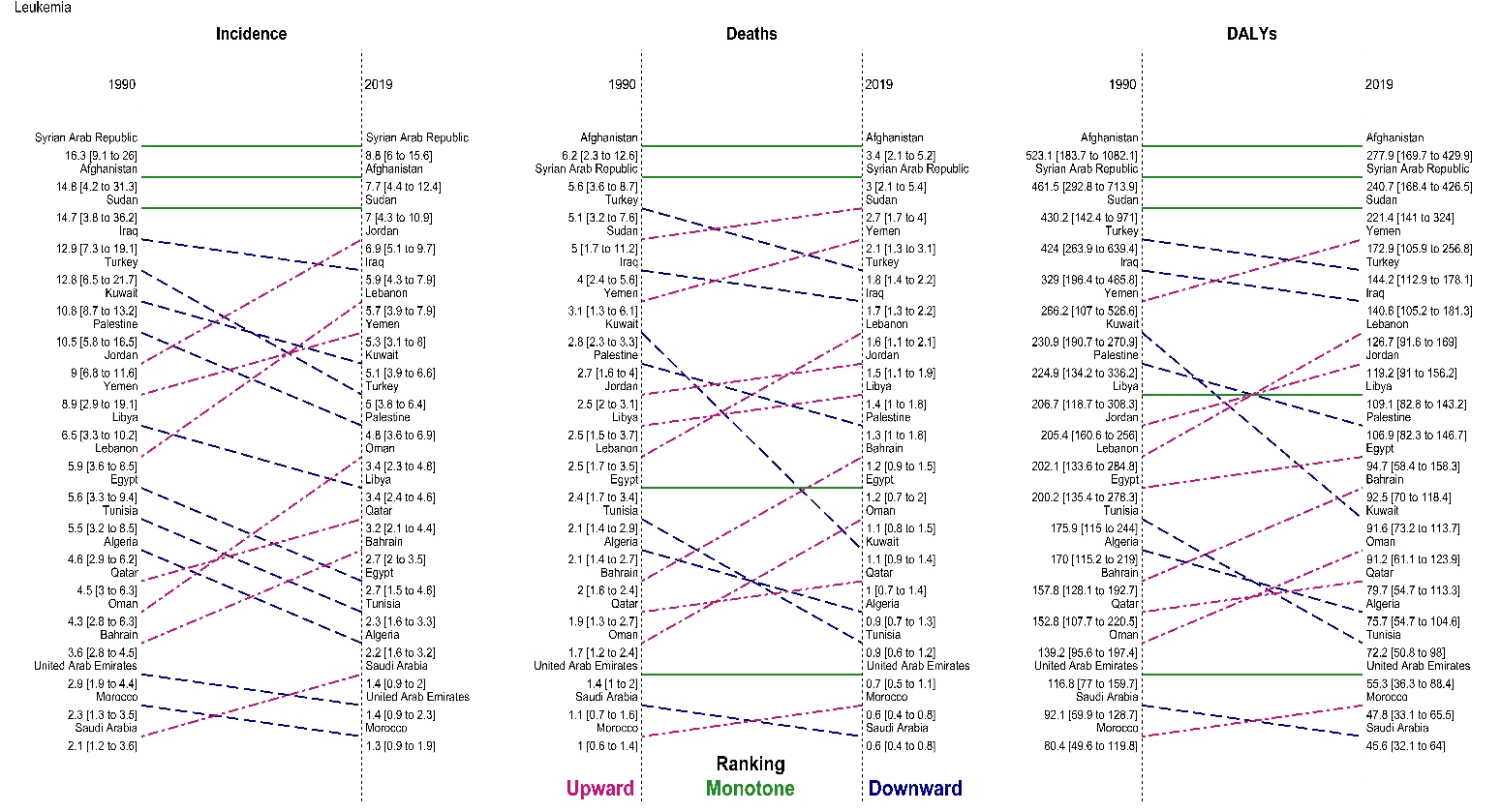


**Supplementary Figure 5:** Arrow chart of the positions of the NAME countries in leukemia incidence, deaths, and DALYs from 1990 to 2019. The results are reported as rates per 100,000.


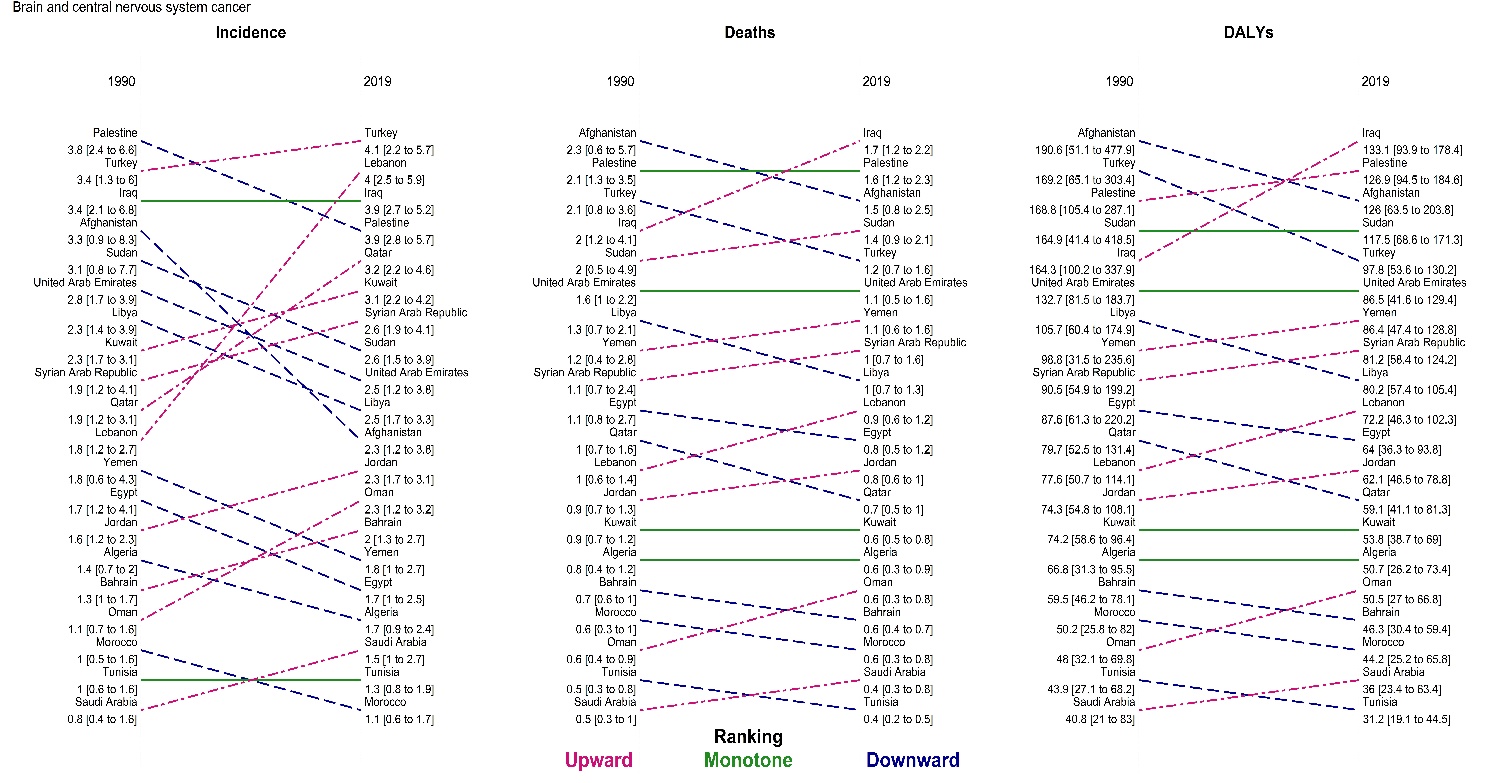


**Supplementary Figure 6:** Arrow chart of the positions of the NAME countries in brain and CNS cancers incidence, deaths, and DALYs from 1990 to 2019. The results are reported as rates per 100,000.


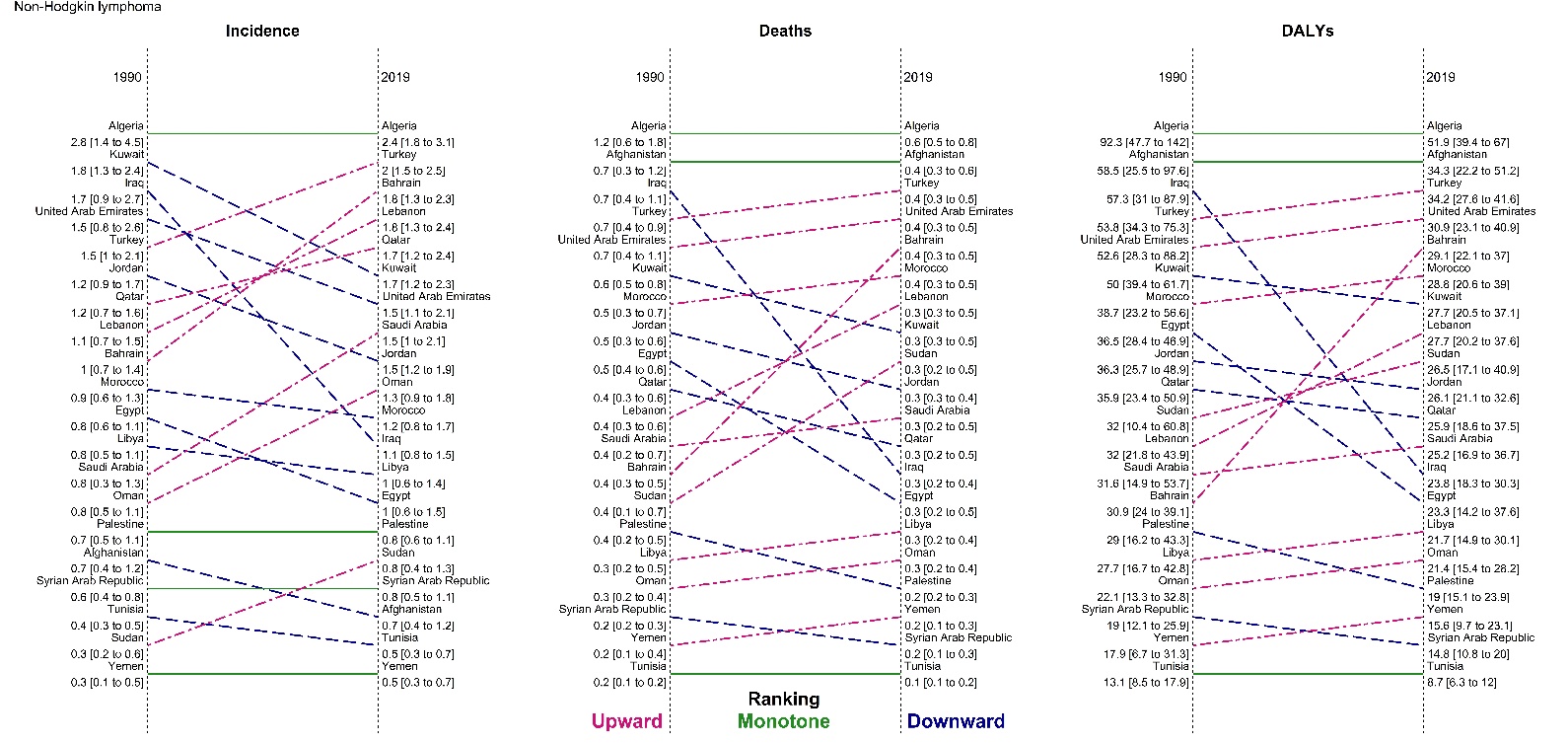


**Supplementary Figure 7:** Arrow chart of the positions of the NAME countries in non-Hodgkin lymphoma incidence, deaths, and DALYs from 1990 to 2019. The results are reported as rates per 100,000.

**Supplementary Table 1:** Details of the countries and the studied cancers.

| Country name | Cause name | level | GBD code |
| --- | --- | --- | --- |
| Afghanistan | **Neoplasms** | 2 | B.1 |
| Algeria | Lip and oral cavity cancer | 3 | B.1.1 |
| Bahrain | Nasopharynx cancer | 3 | B.1.2 |
| Egypt | Stomach cancer | 3 | B.1.5 |
| Iran (the Islamic Republic of) | Colon and rectum cancer | 3 | B.1.6 |
| Iraq | Liver cancer | 3 | B.1.7 |
| Jordan | *Liver cancer due to hepatitis B* | 4 | B.1.7.1 |
| Kuwait | *Liver cancer due to hepatitis C* | 4 | B.1.7.2 |
| Lebanon | *Liver cancer due to alcohol use* | 4 | B.1.7.3 |
| Libya | *Liver cancer due to NASH* | 4 | B.1.7.4 |
| Morocco | *Liver cancer due to other causes* | 4 | B.1.7.5 |
| Oman | Pancreatic cancer | 3 | B.1.9 |
| Palestine | Tracheal, bronchus, and lung cancer | 3 | B.1.11 |
| Qatar | Malignant skin melanoma | 3 | B.1.12 |
| Saudi Arabia | Breast cancer | 3 | B.1.14 |
| Sudan | Cervical cancer | 3 | B.1.15 |
| Syrian Arab Republic | Ovarian cancer | 3 | B.1.17 |
| Tunisia | Testicular cancer | 3 | B.1.19 |
| Turkey | Kidney cancer | 3 | B.1.20 |
| United Arab Emirates | Bladder cancer | 3 | B.1.21 |
| Yemen | Brain and central nervous system cancer | 3 | B.1.22 |
|  | Thyroid cancer | 3 | B.1.23 |
|  | Hodgkin lymphoma | 3 | B.1.25 |
|  | Non-Hodgkin lymphoma | 3 | B.1.26 |
|  | Leukemia | 3 | B.1.28 |
|  | *Acute lymphoid leukemia* | 4 | B.1.28.1 |
|  | *Chronic lymphoid leukemia* | 4 | B.1.28.2 |
|  | *Acute myeloid leukemia* | 4 | B.1.28.3 |
|  | *Chronic myeloid leukemia* | 4 | B.1.28.4 |
|  | *Other leukemia* | 4 | B.1.28.5 |
|  | Other malignant neoplasms | 3 | B.1.29 |
|  | Other neoplasms | 3 | B.1.30 |
|  | *Myelodysplastic, myeloproliferative, and other hematopoietic neoplasms* | 4 | B.1.30.1 |
|  | *Benign and in situ intestinal neoplasms* | 4 | B.1.30.2 |
|  | *Benign and in situ cervical and uterine neoplasms* | 4 | B.1.30.3 |
|  | *Other benign and in situ neoplasms* | 4 | B.1.30.4 |

**Supplementary Table 2:** The incidence, deaths, and DALYs of pediatric cancers overall by number and rate (per 100,000) with percent change in rate from 1990 to 2019 and 95% uncertainty intervals (UI) in the NAME countries, by sex

| Location | Sex | Cause | Incidence | | | DALYs | | | Deaths | | |
| --- | --- | --- | --- | --- | --- | --- | --- | --- | --- | --- | --- |
|  |  |  | Number | Rate per 100,00 | Change in rates (%) | Number | Rate per 100,00 | Change in rates (%) | Number | Rate per 100,00 | Change in rates (%) |
| Afghanistan | Both | Neoplasms | 546,351 (383,957 to 773,473) | 2529 (1777.3 to 3580.3) | -0.7 (-2.4 to 0.8) | 155,049 (112,321 to 208,996) | 717.7 (519.9 to 967.4) | -36.8 (-60.7 to 21) | 1,918 (1,404 to 2,565) | 8.9 (6.5 to 11.9) | -35.7 (-59.5 to 20.3) |
|  |  | Other malignant neoplasms | 1,140 (840 to 1,475) | 5.3 (3.9 to 6.8) | -6 (-46 to 40.8) | 45,546 (33,605 to 59,409) | 210.8 (155.6 to 275) | -22.5 (-57.1 to 20.2) | 567 (422 to 732) | 2.6 (2 to 3.4) | -22.1 (-56 to 18.8) |
|  |  | Other neoplasms | 542,460 (379,856 to 770,239) | 2511 (1758.3 to 3565.4) | -0.3 (-1.8 to 1) | 265 (207 to 342) | 1.2 (1 to 1.6) | -14.8 (-49 to 32.4) | 3 (2 to 4) | 0 (0 to 0) | -15.5 (-52.3 to 39.9) |
|  | Female | Neoplasms | 312,763 (220,789 to 446,090) | 2981.6 (2104.8 to 4252.6) | -0.5 (-2.5 to 1.1) | 86,372 (61,220 to 120,089) | 823.4 (583.6 to 1144.8) | -37.3 (-62.8 to 17.5) | 1,069 (763 to 1,471) | 10.2 (7.3 to 14) | -36.2 (-61.3 to 16.4) |
|  |  | Other malignant neoplasms | 895 (647 to 1,194) | 8.5 (6.2 to 11.4) | -3.7 (-51.6 to 60.7) | 34,142 (24,875 to 46,248) | 325.5 (237.1 to 440.9) | -22.3 (-63 to 32.1) | 423 (310 to 565) | 4 (3 to 5.4) | -22 (-61.7 to 29.9) |
|  |  | Other neoplasms | 310,411 (218,511 to 443,769) | 2959.2 (2083.1 to 4230.5) | -0.2 (-1.8 to 1.3) | 130 (99 to 183) | 1.2 (0.9 to 1.7) | -16.5 (-62.5 to 32) | 1 (1 to 2) | 0 (0 to 0) | -17.3 (-65.7 to 39.9) |
|  | Male | Neoplasms | 233,588 (160,941 to 329,213) | 2101.8 (1448.1 to 2962.3) | -0.4 (-2.2 to 1) | 68,678 (42,483 to 98,837) | 618 (382.3 to 889.3) | -35.9 (-63.6 to 35.3) | 850 (532 to 1,210) | 7.6 (4.8 to 10.9) | -34.8 (-62.4 to 35.4) |
|  |  | Other malignant neoplasms | 244 (153 to 343) | 2.2 (1.4 to 3.1) | -11.2 (-51.6 to 41.5) | 11,403 (7,096 to 16,114) | 102.6 (63.8 to 145) | -21.1 (-58.9 to 30.1) | 145 (89 to 204) | 1.3 (0.8 to 1.8) | -20.6 (-58.1 to 29.5) |
|  |  | Other neoplasms | 232,049 (159,568 to 327,778) | 2088 (1435.8 to 2949.4) | -0.2 (-1.6 to 1.1) | 135 (104 to 180) | 1.2 (0.9 to 1.6) | -13.1 (-55.6 to 52.9) | 1 (1 to 2) | 0 (0 to 0) | -13.8 (-57.5 to 59.2) |
| Algeria | Both | Neoplasms | 377,057 (263,873 to 534,883) | 2551.3 (1785.5 to 3619.2) | -0.6 (-1 to -0.2) | 46,776 (38,753 to 57,253) | 316.5 (262.2 to 387.4) | -42.6 (-55.5 to -23.5) | 580 (480 to 708) | 3.9 (3.2 to 4.8) | -43 (-55.8 to -24) |
|  |  | Other malignant neoplasms | 705 (550 to 890) | 4.8 (3.7 to 6) | 11.2 (-18.9 to 53.9) | 13,916 (10,965 to 17,663) | 94.2 (74.2 to 119.5) | -32.4 (-51.5 to 0) | 173 (136 to 221) | 1.2 (0.9 to 1.5) | -32.9 (-51.5 to -0.3) |
|  |  | Other neoplasms | 374,888 (261,738 to 532,904) | 2536.6 (1771 to 3605.8) | -0.6 (-0.9 to -0.2) | 182 (149 to 223) | 1.2 (1 to 1.5) | -9.9 (-33.7 to 15.8) | 2 (1 to 2) | 0 (0 to 0) | -13.4 (-40.1 to 18.8) |
|  | Female | Neoplasms | 216,280 (152,008 to 309,218) | 3000.1 (2108.6 to 4289.3) | -0.4 (-0.8 to 0.1) | 20,588 (16,217 to 25,564) | 285.6 (225 to 354.6) | -37.8 (-52.3 to -17.9) | 254 (200 to 314) | 3.5 (2.8 to 4.3) | -38.3 (-52.5 to -18.7) |
|  |  | Other malignant neoplasms | 422 (323 to 539) | 5.9 (4.5 to 7.5) | 32.1 (-9.4 to 89.1) | 6,294 (4,999 to 7,851) | 87.3 (69.3 to 108.9) | -28.6 (-49.7 to 3.2) | 77 (61 to 97) | 1.1 (0.9 to 1.3) | -29.5 (-50.3 to 2) |
|  |  | Other neoplasms | 215,177 (150,868 to 308,168) | 2984.8 (2092.8 to 4274.7) | -0.4 (-0.7 to 0) | 91 (75 to 112) | 1.3 (1 to 1.6) | -8.7 (-34.5 to 21) | 1 (1 to 1) | 0 (0 to 0) | -12.1 (-40.9 to 24.2) |
|  | Male | Neoplasms | 160,777 (110,892 to 227,412) | 2123.9 (1464.9 to 3004.2) | -0.7 (-1.1 to -0.2) | 26,188 (20,867 to 33,113) | 346 (275.7 to 437.4) | -45.9 (-60.5 to -20.6) | 326 (260 to 409) | 4.3 (3.4 to 5.4) | -46.3 (-60.7 to -21.6) |
|  |  | Other malignant neoplasms | 282 (196 to 414) | 3.7 (2.6 to 5.5) | -10.1 (-43.5 to 43.3) | 7,622 (5,450 to 10,824) | 100.7 (72 to 143) | -35.4 (-59.3 to 9.4) | 96 (69 to 136) | 1.3 (0.9 to 1.8) | -35.4 (-59.1 to 9.3) |
|  |  | Other neoplasms | 159,711 (109,835 to 226,214) | 2109.8 (1450.9 to 2988.3) | -0.6 (-0.9 to -0.1) | 91 (73 to 117) | 1.2 (1 to 1.5) | -11 (-40.8 to 21.6) | 1 (1 to 1) | 0 (0 to 0) | -14.7 (-46.3 to 24.5) |
| Bahrain | Both | Neoplasms | 8,560 (5,935 to 11,989) | 2668.4 (1850.1 to 3737.4) | 6.5 (1 to 13.2) | 871 (730 to 1,019) | 271.4 (227.4 to 317.6) | -30.8 (-43.7 to -15.2) | 11 (9 to 13) | 3.4 (2.9 to 4) | -30.6 (-43.7 to -14.9) |
|  |  | Other malignant neoplasms | 17 (14 to 21) | 5.3 (4.3 to 6.5) | 54.8 (19.7 to 108.4) | 223 (183 to 278) | 69.4 (57.1 to 86.5) | -27.1 (-42.4 to -0.8) | 3 (2 to 3) | 0.9 (0.7 to 1.1) | -27.1 (-43 to -0.7) |
|  |  | Other neoplasms | 8,512 (5,889 to 11,937) | 2653.4 (1835.6 to 3721.1) | 6.4 (0.9 to 13.1) | 4 (3 to 6) | 1.3 (1 to 1.7) | -58.1 (-69.6 to -37.2) | 0 (0 to 0) | 0 (0 to 0) | -63.5 (-74.3 to -41.8) |
|  | Female | Neoplasms | 4,876 (3,383 to 6,973) | 3113.8 (2160.4 to 4452.8) | 5.3 (0.1 to 11.7) | 341 (288 to 402) | 218 (184 to 257) | -33.9 (-46.1 to -16.3) | 4 (4 to 5) | 2.7 (2.3 to 3.2) | -34.6 (-46.7 to -17.1) |
|  |  | Other malignant neoplasms | 11 (8 to 14) | 6.7 (5.1 to 8.8) | 77.8 (28.8 to 155.7) | 90 (73 to 110) | 57.3 (46.7 to 70) | -32.3 (-48.1 to -9.7) | 1 (1 to 1) | 0.7 (0.6 to 0.9) | -34.2 (-49.7 to -11) |
|  |  | Other neoplasms | 4,852 (3,361 to 6,949) | 3098.5 (2146.5 to 4437.8) | 5.2 (0 to 11.6) | 2 (2 to 2) | 1.2 (1 to 1.5) | -68.5 (-78.2 to -51.8) | 0 (0 to 0) | 0 (0 to 0) | -74.3 (-82.7 to -59.1) |
|  | Male | Neoplasms | 3,684 (2,500 to 5,135) | 2243.7 (1522.2 to 3127) | 8.2 (2 to 15.8) | 529 (407 to 650) | 322.4 (248.1 to 395.7) | -28.7 (-46.2 to -7.9) | 7 (5 to 8) | 4.1 (3.1 to 5) | -27.8 (-45.7 to -6.6) |
|  |  | Other malignant neoplasms | 7 (5 to 9) | 4 (3 to 5.3) | 28.1 (-9.2 to 86.9) | 133 (101 to 179) | 81 (61.7 to 109.2) | -23.1 (-45 to 13.2) | 2 (1 to 2) | 1 (0.8 to 1.4) | -21.6 (-44.5 to 15.1) |
|  |  | Other neoplasms | 3,660 (2,472 to 5,107) | 2228.9 (1505.7 to 3110.3) | 8.1 (2 to 15.7) | 2 (2 to 4) | 1.3 (1 to 2.1) | -41.8 (-61.6 to -5.3) | 0 (0 to 0) | 0 (0 to 0) | -45.7 (-66.7 to -1.9) |
| Egypt | Both | Neoplasms | 1,083,620 (753,572 to 1,542,397) | 2585.4 (1797.9 to 3680) | 2.1 (0.4 to 3.5) | 142,428 (105,428 to 189,458) | 339.8 (251.5 to 452) | -41.8 (-60.9 to -13.2) | 1,769 (1,317 to 2,351) | 4.2 (3.1 to 5.6) | -40.7 (-60 to -12.5) |
|  |  | Other malignant neoplasms | 1,750 (1,237 to 2,367) | 4.2 (3 to 5.6) | 14.6 (-18.6 to 60.2) | 42,852 (30,250 to 59,693) | 102.2 (72.2 to 142.4) | -37.2 (-57.7 to -10.1) | 529 (374 to 740) | 1.3 (0.9 to 1.8) | -36.3 (-56.7 to -9) |
|  |  | Other neoplasms | 1,078,481 (748,248 to 1,536,920) | 2573.1 (1785.2 to 3666.9) | 2.2 (0.6 to 3.6) | 382 (280 to 493) | 0.9 (0.7 to 1.2) | -24.3 (-49 to 8.5) | 4 (2 to 5) | 0 (0 to 0) | -27.4 (-55.3 to 12.5) |
|  | Female | Neoplasms | 613,158 (428,339 to 878,259) | 3035.4 (2120.5 to 4347.8) | 2 (0.2 to 3.3) | 57,159 (42,939 to 74,392) | 283 (212.6 to 368.3) | -46.5 (-66.4 to -19.8) | 706 (530 to 918) | 3.5 (2.6 to 4.5) | -45.7 (-65.1 to -19.3) |
|  |  | Other malignant neoplasms | 1,026 (674 to 1,465) | 5.1 (3.3 to 7.3) | 23.7 (-26.7 to 97) | 19,382 (12,928 to 28,184) | 95.9 (64 to 139.5) | -41.1 (-66.2 to -6.7) | 237 (159 to 344) | 1.2 (0.8 to 1.7) | -40.8 (-65.5 to -6.8) |
|  |  | Other neoplasms | 610,708 (425,929 to 875,883) | 3023.3 (2108.6 to 4336) | 2.1 (0.4 to 3.4) | 184 (129 to 242) | 0.9 (0.6 to 1.2) | -29.4 (-64.3 to 8.4) | 2 (1 to 2) | 0 (0 to 0) | -33.4 (-69 to 14.2) |
|  | Male | Neoplasms | 470,463 (323,509 to 667,782) | 2166.7 (1489.9 to 3075.5) | 2.5 (0.8 to 3.9) | 85,269 (58,341 to 121,217) | 392.7 (268.7 to 558.3) | -38.1 (-60.9 to -1) | 1,064 (734 to 1,506) | 4.9 (3.4 to 6.9) | -36.8 (-59.7 to 0.4) |
|  |  | Other malignant neoplasms | 724 (450 to 1,137) | 3.3 (2.1 to 5.2) | 3.8 (-36.1 to 61.5) | 23,470 (15,014 to 35,797) | 108.1 (69.1 to 164.9) | -33.5 (-60.2 to 8.1) | 293 (189 to 446) | 1.3 (0.9 to 2.1) | -32.1 (-58.5 to 10.1) |
|  |  | Other neoplasms | 467,773 (319,729 to 664,773) | 2154.4 (1472.5 to 3061.7) | 2.6 (1 to 4) | 198 (146 to 261) | 0.9 (0.7 to 1.2) | -18.8 (-50.1 to 22) | 2 (1 to 3) | 0 (0 to 0) | -21.2 (-55.3 to 28.3) |
| Iran (Islamic republic of) | Both | Neoplasms | 655,307 (446,341 to 922,682) | 2534.1 (1726 to 3568) | 1.3 (-0.1 to 3) | 112,025 (78,319 to 134,561) | 433.2 (302.9 to 520.4) | -39.2 (-57 to -14.1) | 1,388 (980 to 1,661) | 5.4 (3.8 to 6.4) | -38.1 (-55.8 to -13.7) |
|  |  | Other malignant neoplasms | 1,867 (1,369 to 2,298) | 7.2 (5.3 to 8.9) | 91.5 (30.9 to 156.3) | 23,232 (16,852 to 27,916) | 89.8 (65.2 to 108) | -12.5 (-45 to 20.6) | 281 (207 to 333) | 1.1 (0.8 to 1.3) | -13.1 (-44.4 to 18.1) |
|  |  | Other neoplasms | 649,346 (440,364 to 916,334) | 2511 (1702.9 to 3543.5) | 1.3 (0 to 3.1) | 320 (261 to 393) | 1.2 (1 to 1.5) | -18.3 (-37.3 to 5.8) | 3 (2 to 3) | 0 (0 to 0) | -19.6 (-44.8 to 13.8) |
|  | Female | Neoplasms | 357,931 (243,171 to 505,724) | 2840.9 (1930 to 4013.9) | 1.1 (-0.3 to 2.9) | 51,679 (37,126 to 60,578) | 410.2 (294.7 to 480.8) | -41 (-58.5 to -12.2) | 636 (461 to 739) | 5 (3.7 to 5.9) | -39.9 (-57.2 to -11.2) |
|  |  | Other malignant neoplasms | 1,341 (950 to 1,737) | 10.6 (7.5 to 13.8) | 130.9 (56.5 to 232.3) | 11,495 (8,466 to 13,725) | 91.2 (67.2 to 108.9) | -7.4 (-42.9 to 27.5) | 136 (100 to 161) | 1.1 (0.8 to 1.3) | -9.3 (-42.5 to 23.2) |
|  |  | Other neoplasms | 354,710 (240,497 to 502,520) | 2815.3 (1908.8 to 3988.5) | 1.1 (-0.2 to 3) | 157 (126 to 198) | 1.2 (1 to 1.6) | -22.4 (-42.2 to -0.6) | 1 (1 to 2) | 0 (0 to 0) | -21.7 (-47.4 to 10.2) |
|  | Male | Neoplasms | 297,376 (201,440 to 423,573) | 2242.6 (1519.1 to 3194.3) | 1.6 (0.1 to 3.2) | 60,346 (38,889 to 75,993) | 455.1 (293.3 to 573.1) | -37.5 (-60.5 to -2.6) | 752 (491 to 941) | 5.7 (3.7 to 7.1) | -36.5 (-59.2 to -3) |
|  |  | Other malignant neoplasms | 526 (363 to 699) | 4 (2.7 to 5.3) | 33.6 (-28 to 111.3) | 11,736 (8,301 to 15,056) | 88.5 (62.6 to 113.5) | -17 (-56.7 to 32.6) | 145 (104 to 184) | 1.1 (0.8 to 1.4) | -16.4 (-55.5 to 31.4) |
|  |  | Other neoplasms | 294,635 (198,616 to 421,098) | 2221.9 (1497.8 to 3175.6) | 1.6 (0.2 to 3.2) | 163 (132 to 200) | 1.2 (1 to 1.5) | -13.9 (-37.2 to 20.9) | 1 (1 to 2) | 0 (0 to 0) | -17.5 (-45.2 to 29.2) |
| Iraq | Both | Neoplasms | 475,658 (331,951 to 669,438) | 2614.9 (1824.9 to 3680.2) | 3.5 (0.3 to 7.4) | 78,954 (63,352 to 98,463) | 434 (348.3 to 541.3) | -40.2 (-58.1 to -7.1) | 983 (788 to 1,224) | 5.4 (4.3 to 6.7) | -39 (-57.1 to -5.5) |
|  |  | Other malignant neoplasms | 795 (601 to 1,049) | 4.4 (3.3 to 5.8) | 21.3 (-9.2 to 69) | 16,636 (12,521 to 23,271) | 91.5 (68.8 to 127.9) | -23.1 (-43.8 to 5.4) | 208 (156 to 294) | 1.1 (0.9 to 1.6) | -21.9 (-42.6 to 6.9) |
|  |  | Other neoplasms | 472,312 (329,412 to 665,930) | 2596.5 (1810.9 to 3660.9) | 3.8 (0.5 to 7.6) | 176 (142 to 218) | 1 (0.8 to 1.2) | -16.6 (-36.2 to 3.5) | 2 (1 to 2) | 0 (0 to 0) | -20.6 (-42.2 to 3.7) |
|  | Female | Neoplasms | 271,102 (189,598 to 386,364) | 3062.3 (2141.7 to 4364.3) | 3.1 (-0.1 to 6.8) | 37,101 (29,680 to 46,356) | 419.1 (335.3 to 523.6) | -36.5 (-58.5 to -1) | 460 (368 to 571) | 5.2 (4.2 to 6.5) | -35.3 (-57.5 to -0.9) |
|  |  | Other malignant neoplasms | 469 (353 to 621) | 5.3 (4 to 7) | 39.4 (-1.3 to 101.8) | 7,775 (5,885 to 10,365) | 87.8 (66.5 to 117.1) | -20.2 (-42.7 to 13.1) | 96 (73 to 128) | 1.1 (0.8 to 1.4) | -19.9 (-42.3 to 12.7) |
|  |  | Other neoplasms | 269,384 (187,671 to 384,887) | 3042.9 (2119.9 to 4347.6) | 3.2 (0 to 7.1) | 87 (69 to 110) | 1 (0.8 to 1.2) | -22.3 (-47.1 to 3.8) | 1 (1 to 1) | 0 (0 to 0) | -27 (-53.1 to 3.3) |
|  | Male | Neoplasms | 204,556 (140,553 to 288,181) | 2190.7 (1505.2 to 3086.2) | 4.3 (0.9 to 8.5) | 41,852 (30,715 to 54,729) | 448.2 (328.9 to 586.1) | -43.1 (-64.9 to -2.9) | 523 (382 to 682) | 5.6 (4.1 to 7.3) | -41.9 (-63.6 to -2) |
|  |  | Other malignant neoplasms | 327 (223 to 507) | 3.5 (2.4 to 5.4) | 2.3 (-34.4 to 56.2) | 8,861 (6,104 to 14,024) | 94.9 (65.4 to 150.2) | -25.5 (-56.4 to 16) | 112 (77 to 178) | 1.2 (0.8 to 1.9) | -23.7 (-55.1 to 17.7) |
|  |  | Other neoplasms | 202,928 (139,177 to 286,450) | 2173.2 (1490.5 to 3067.7) | 4.7 (1.3 to 8.6) | 88 (70 to 111) | 0.9 (0.8 to 1.2) | -10 (-28.9 to 11) | 1 (1 to 1) | 0 (0 to 0) | -13.1 (-34.5 to 11) |
| Jordan | Both | Neoplasms | 100,578 (72,077 to 137,218) | 2047.9 (1467.6 to 2793.9) | 7.5 (-4.8 to 22.3) | 15,928 (13,225 to 19,652) | 324.3 (269.3 to 400.1) | -26.4 (-41.5 to -6.4) | 197 (164 to 243) | 4 (3.3 to 4.9) | -26.9 (-41.8 to -6.9) |
|  |  | Other malignant neoplasms | 260 (198 to 347) | 5.3 (4 to 7.1) | 66.9 (26.1 to 123.4) | 4,067 (3,130 to 5,635) | 82.8 (63.7 to 114.7) | -9.6 (-32.9 to 21.1) | 51 (39 to 70) | 1 (0.8 to 1.4) | -10.5 (-33.4 to 20.2) |
|  |  | Other neoplasms | 99,632 (71,154 to 136,331) | 2028.6 (1448.8 to 2775.8) | 7.4 (-4.8 to 22.3) | 51 (41 to 62) | 1 (0.8 to 1.3) | 9.7 (-10.4 to 36.5) | 1 (0 to 1) | 0 (0 to 0) | 8.9 (-12.9 to 38.9) |
|  | Female | Neoplasms | 53,777 (38,887 to 74,013) | 2263 (1636.4 to 3114.5) | 4.5 (-10 to 25.2) | 7,196 (5,866 to 8,896) | 302.8 (246.9 to 374.3) | -30.9 (-47 to -5.6) | 88 (72 to 109) | 3.7 (3 to 4.6) | -31.4 (-47.5 to -6.7) |
|  |  | Other malignant neoplasms | 159 (116 to 216) | 6.7 (4.9 to 9.1) | 83.9 (29.2 to 166) | 1,822 (1,365 to 2,502) | 76.7 (57.5 to 105.3) | -14.6 (-40.4 to 22) | 22 (16 to 31) | 0.9 (0.7 to 1.3) | -16.4 (-41.8 to 19.7) |
|  |  | Other neoplasms | 53,290 (38,412 to 73,458) | 2242.5 (1616.4 to 3091.1) | 4.5 (-10.1 to 25.4) | 23 (19 to 28) | 1 (0.8 to 1.2) | 3.2 (-17.2 to 29.5) | 0 (0 to 0) | 0 (0 to 0) | 1.7 (-20 to 32.9) |
|  | Male | Neoplasms | 46,801 (33,156 to 64,679) | 1846.2 (1307.9 to 2551.5) | 11.1 (-2.7 to 25.6) | 8,732 (7,020 to 11,257) | 344.5 (276.9 to 444.1) | -22.4 (-41 to 3.2) | 109 (87 to 140) | 4.3 (3.4 to 5.5) | -22.7 (-41.3 to 1.7) |
|  |  | Other malignant neoplasms | 101 (71 to 143) | 4 (2.8 to 5.7) | 45.6 (4.6 to 98.9) | 2,245 (1,621 to 3,284) | 88.6 (63.9 to 129.6) | -5.2 (-33.3 to 30.7) | 28 (21 to 42) | 1.1 (0.8 to 1.6) | -5.1 (-33.6 to 31.3) |
|  |  | Other neoplasms | 46,342 (32,651 to 64,189) | 1828.1 (1288.1 to 2532.2) | 11 (-2.9 to 25.6) | 28 (21 to 35) | 1.1 (0.8 to 1.4) | 15.9 (-13.7 to 54.2) | 0 (0 to 0) | 0 (0 to 0) | 15.6 (-16.6 to 55.7) |
| Kuwait | Both | Neoplasms | 28,286 (19,750 to 40,178) | 2579.9 (1801.4 to 3664.6) | 1.3 (-0.3 to 3.2) | 2,968 (2,485 to 3,529) | 270.7 (226.6 to 321.9) | -48.9 (-58.8 to -36.3) | 36 (30 to 42) | 3.2 (2.7 to 3.9) | -49.2 (-59 to -36.8) |
|  |  | Other malignant neoplasms | 75 (58 to 98) | 6.8 (5.3 to 8.9) | 0.4 (-26.9 to 49.8) | 680 (562 to 816) | 62.1 (51.2 to 74.4) | -46.2 (-57.9 to -27.6) | 8 (7 to 10) | 0.7 (0.6 to 0.9) | -46.8 (-58.2 to -28.4) |
|  |  | Other neoplasms | 28,047 (19,487 to 39,897) | 2558.2 (1777.4 to 3639) | 1.5 (0 to 3.4) | 13 (11 to 17) | 1.2 (1 to 1.5) | -10.4 (-27.6 to 9.6) | 0 (0 to 0) | 0 (0 to 0) | -13.6 (-32.2 to 8) |
|  | Female | Neoplasms | 16,116 (11,314 to 23,038) | 3031.9 (2128.7 to 4334.4) | 1 (-0.2 to 2.5) | 1,216 (1,019 to 1,424) | 228.8 (191.8 to 268) | -52.5 (-62 to -41.5) | 14 (12 to 17) | 2.7 (2.3 to 3.2) | -52.9 (-62.2 to -42.2) |
|  |  | Other malignant neoplasms | 52 (39 to 72) | 9.8 (7.3 to 13.5) | 3.1 (-29.7 to 71.5) | 303 (245 to 380) | 56.9 (46.1 to 71.6) | -51.1 (-62.6 to -26.7) | 3 (3 to 4) | 0.7 (0.5 to 0.8) | -52.9 (-63.7 to -30.9) |
|  |  | Other neoplasms | 15,993 (11,178 to 22,905) | 3008.9 (2102.9 to 4309.3) | 1.3 (0 to 2.8) | 7 (5 to 9) | 1.3 (1 to 1.6) | -12.4 (-33.5 to 16.4) | 0 (0 to 0) | 0 (0 to 0) | -17.2 (-34.3 to 0.9) |
|  | Male | Neoplasms | 12,170 (8,394 to 17,251) | 2154.6 (1486 to 3054.2) | 2.4 (0.4 to 4.8) | 1,752 (1,404 to 2,171) | 310.1 (248.6 to 384.3) | -46.1 (-58.8 to -29.4) | 21 (17 to 26) | 3.8 (3 to 4.6) | -46.4 (-59 to -29.8) |
|  |  | Other malignant neoplasms | 23 (17 to 29) | 4 (3.1 to 5.1) | -3.2 (-29.1 to 35.2) | 378 (296 to 465) | 66.9 (52.5 to 82.3) | -41.5 (-55.9 to -19.7) | 5 (4 to 6) | 0.8 (0.6 to 1) | -41.2 (-55.9 to -19.4) |
|  |  | Other neoplasms | 12,054 (8,284 to 17,100) | 2134 (1466.6 to 3027.4) | 2.5 (0.6 to 4.9) | 7 (5 to 8) | 1.2 (1 to 1.5) | -8.2 (-27.1 to 13.8) | 0 (0 to 0) | 0 (0 to 0) | -10.3 (-34.8 to 18.3) |
| Lebanon | Both | Neoplasms | 43,964 (30,871 to 62,434) | 2544.7 (1786.9 to 3613.8) | 1.2 (0.1 to 2.1) | 6,904 (5,301 to 8,629) | 399.6 (306.8 to 499.4) | -15.8 (-35.3 to 14.2) | 85 (66 to 106) | 4.9 (3.8 to 6.1) | -16.7 (-35.8 to 10.5) |
|  |  | Other malignant neoplasms | 150 (101 to 209) | 8.7 (5.9 to 12.1) | 142.8 (75.3 to 238.8) | 2,122 (1,418 to 3,033) | 122.8 (82.1 to 175.6) | 20.1 (-13.6 to 67.2) | 26 (18 to 37) | 1.5 (1 to 2.2) | 18.8 (-14.5 to 64.8) |
|  |  | Other neoplasms | 43,486 (30,345 to 61,956) | 2517 (1756.4 to 3586.2) | 0.7 (-0.3 to 1.6) | 20 (15 to 26) | 1.2 (0.9 to 1.5) | -1.9 (-26.9 to 26.2) | 0 (0 to 0) | 0 (0 to 0) | -3.4 (-34.9 to 35.9) |
|  | Female | Neoplasms | 24,707 (17,423 to 35,500) | 2992.7 (2110.4 to 4300) | 1.1 (0 to 2) | 2,759 (2,055 to 3,588) | 334.2 (248.9 to 434.6) | -18.8 (-41.8 to 20.3) | 34 (25 to 44) | 4.1 (3.1 to 5.3) | -19.9 (-42.3 to 16.8) |
|  |  | Other malignant neoplasms | 82 (55 to 116) | 10 (6.7 to 14) | 165.3 (79.2 to 296.7) | 809 (539 to 1,131) | 98 (65.2 to 137) | 10.6 (-26.8 to 57.1) | 10 (7 to 14) | 1.2 (0.8 to 1.7) | 8 (-28.3 to 55.3) |
|  |  | Other neoplasms | 24,490 (17,189 to 35,241) | 2966.5 (2082.1 to 4268.8) | 0.7 (-0.4 to 1.7) | 10 (7 to 13) | 1.2 (0.9 to 1.6) | -2.6 (-31.9 to 32.8) | 0 (0 to 0) | 0 (0 to 0) | -3.9 (-40.2 to 45.1) |
|  | Male | Neoplasms | 19,257 (13,257 to 27,172) | 2134.7 (1469.6 to 3012.1) | 1.2 (0.3 to 2.1) | 4,144 (3,160 to 5,321) | 459.4 (350.3 to 589.9) | -13.6 (-37.1 to 20.5) | 51 (39 to 66) | 5.7 (4.3 to 7.3) | -14.4 (-37.9 to 18.8) |
|  |  | Other malignant neoplasms | 68 (43 to 102) | 7.5 (4.7 to 11.4) | 120.2 (47.4 to 238.7) | 1,313 (832 to 1,957) | 145.5 (92.2 to 216.9) | 26.9 (-16.6 to 90.4) | 17 (11 to 25) | 1.8 (1.2 to 2.8) | 26.4 (-17.3 to 90.4) |
|  |  | Other neoplasms | 18,995 (13,012 to 26,939) | 2105.7 (1442.4 to 2986.3) | 0.6 (-0.3 to 1.5) | 10 (8 to 14) | 1.2 (0.8 to 1.5) | -1.2 (-28.1 to 30.6) | 0 (0 to 0) | 0 (0 to 0) | -3 (-35.7 to 37) |
| Libya | Both | Neoplasms | 55,498 (38,399 to 77,749) | 2699 (1867.4 to 3781) | 5.6 (0.1 to 12.1) | 7,074 (5,842 to 8,711) | 344 (284.1 to 423.6) | -31.2 (-51.1 to 3.5) | 89 (74 to 109) | 4.3 (3.6 to 5.3) | -29.5 (-49.7 to 4.6) |
|  |  | Other malignant neoplasms | 77 (58 to 111) | 3.8 (2.8 to 5.4) | 29.8 (-1.5 to 77.4) | 1,688 (1,253 to 2,518) | 82.1 (60.9 to 122.4) | -14.4 (-37.8 to 19.6) | 21 (16 to 32) | 1 (0.8 to 1.5) | -12.7 (-36.2 to 21) |
|  |  | Other neoplasms | 55,211 (38,143 to 77,492) | 2685 (1854.9 to 3768.5) | 5.6 (0.2 to 12.3) | 22 (18 to 28) | 1.1 (0.9 to 1.3) | -19 (-41.9 to 6.3) | 0 (0 to 0) | 0 (0 to 0) | -21.3 (-47.3 to 12.2) |
|  | Female | Neoplasms | 31,510 (21,659 to 45,093) | 3148.1 (2163.9 to 4505.2) | 5.3 (-0.3 to 12.4) | 3,515 (2,848 to 4,344) | 351.2 (284.5 to 434) | -31 (-52.9 to 6.2) | 44 (36 to 55) | 4.4 (3.6 to 5.5) | -29.3 (-51.6 to 7.7) |
|  |  | Other malignant neoplasms | 48 (36 to 67) | 4.8 (3.6 to 6.7) | 41.8 (-1 to 107.7) | 897 (645 to 1,251) | 89.6 (64.4 to 124.9) | -10.6 (-40 to 30.9) | 11 (8 to 16) | 1.1 (0.8 to 1.6) | -9.2 (-39 to 33.8) |
|  |  | Other neoplasms | 31,350 (21,501 to 44,902) | 3132.2 (2148.2 to 4486.1) | 5.4 (-0.3 to 12.6) | 12 (9 to 15) | 1.2 (0.9 to 1.5) | -8.5 (-34.4 to 22.2) | 0 (0 to 0) | 0 (0 to 0) | -8.9 (-40.5 to 32.4) |
|  | Male | Neoplasms | 23,989 (16,166 to 33,312) | 2272.9 (1531.8 to 3156.3) | 7.1 (1.8 to 13) | 3,559 (2,815 to 4,633) | 337.2 (266.7 to 439) | -31.3 (-54.8 to 8.1) | 45 (36 to 58) | 4.3 (3.4 to 5.5) | -29.7 (-53.1 to 10.5) |
|  |  | Other malignant neoplasms | 29 (19 to 48) | 2.7 (1.8 to 4.6) | 14.9 (-22.8 to 76.2) | 792 (544 to 1,325) | 75 (51.6 to 125.5) | -18.1 (-44.3 to 26.9) | 10 (7 to 17) | 1 (0.7 to 1.6) | -16.1 (-42.8 to 30.1) |
|  |  | Other neoplasms | 23,861 (16,044 to 33,167) | 2260.9 (1520.2 to 3142.6) | 7.1 (2 to 13.1) | 10 (8 to 13) | 1 (0.8 to 1.3) | -28.4 (-58.5 to 7.2) | 0 (0 to 0) | 0 (0 to 0) | -31.6 (-63 to 13.6) |
| Morocco | Both | Neoplasms | 329,511 (229,586 to 464,257) | 2627 (1830.3 to 3701.2) | 2.4 (0.3 to 4.8) | 28,264 (22,227 to 35,658) | 225.3 (177.2 to 284.3) | -23.2 (-47.7 to 13.7) | 355 (280 to 444) | 2.8 (2.2 to 3.5) | -22 (-46.8 to 13.6) |
|  |  | Other malignant neoplasms | 323 (233 to 427) | 2.6 (1.9 to 3.4) | 38.6 (-4.3 to 107.9) | 7,917 (5,755 to 10,367) | 63.1 (45.9 to 82.7) | -15.8 (-44.6 to 28.3) | 99 (72 to 130) | 0.8 (0.6 to 1) | -14.6 (-43 to 28.3) |
|  |  | Other neoplasms | 328,408 (228,560 to 462,915) | 2618.2 (1822.1 to 3690.5) | 2.4 (0.2 to 4.8) | 126 (92 to 163) | 1 (0.7 to 1.3) | -2.3 (-30.2 to 31.5) | 1 (1 to 2) | 0 (0 to 0) | -1.6 (-35.2 to 44.1) |
|  | Female | Neoplasms | 188,410 (131,284 to 269,279) | 3076.1 (2143.4 to 4396.4) | 2.2 (0.1 to 4.6) | 14,532 (11,214 to 19,278) | 237.3 (183.1 to 314.7) | -16.8 (-45.1 to 24) | 182 (140 to 241) | 3 (2.3 to 3.9) | -15.9 (-43.9 to 25) |
|  |  | Other malignant neoplasms | 200 (138 to 286) | 3.3 (2.2 to 4.7) | 63.6 (-1.2 to 165.6) | 3,529 (2,440 to 5,212) | 57.6 (39.8 to 85.1) | -12 (-45.7 to 42.7) | 44 (30 to 65) | 0.7 (0.5 to 1.1) | -11.5 (-44 to 42.3) |
|  |  | Other neoplasms | 187,736 (130,446 to 268,579) | 3065.1 (2129.7 to 4384.9) | 2.2 (0 to 4.5) | 61 (44 to 81) | 1 (0.7 to 1.3) | -6.6 (-42.7 to 32.5) | 1 (0 to 1) | 0 (0 to 0) | -6.6 (-48 to 47.6) |
|  | Male | Neoplasms | 141,102 (96,498 to 198,980) | 2198.4 (1503.4 to 3100.1) | 3.3 (1 to 5.9) | 13,732 (10,354 to 17,867) | 213.9 (161.3 to 278.4) | -29 (-56 to 18.2) | 173 (131 to 224) | 2.7 (2 to 3.5) | -27.6 (-54.5 to 18.8) |
|  |  | Other malignant neoplasms | 122 (83 to 177) | 1.9 (1.3 to 2.8) | 11.1 (-41 to 92.1) | 4,387 (2,985 to 6,254) | 68.4 (46.5 to 97.4) | -18.9 (-58.2 to 42.9) | 55 (37 to 78) | 0.9 (0.6 to 1.2) | -17.2 (-56.1 to 42.5) |
|  |  | Other neoplasms | 140,671 (96,138 to 198,442) | 2191.7 (1497.8 to 3091.8) | 3.3 (1.1 to 5.9) | 66 (46 to 88) | 1 (0.7 to 1.4) | 2.2 (-33.1 to 45.6) | 1 (0 to 1) | 0 (0 to 0) | 3.1 (-37.7 to 61.4) |
| Oman | Both | Neoplasms | 32,478 (22,809 to 46,121) | 2531.9 (1778.1 to 3595.5) | 1.9 (-0.3 to 4.9) | 3,701 (2,781 to 4,480) | 288.5 (216.8 to 349.2) | -15.3 (-36.5 to 16.1) | 45 (34 to 55) | 3.5 (2.7 to 4.3) | -15.7 (-36.6 to 14.4) |
|  |  | Other malignant neoplasms | 101 (79 to 129) | 7.9 (6.1 to 10.1) | 98.3 (43.8 to 176.4) | 1,103 (897 to 1,344) | 86 (69.9 to 104.8) | -8.1 (-31.3 to 23.6) | 13 (11 to 16) | 1 (0.9 to 1.3) | -9.3 (-31.5 to 21.2) |
|  |  | Other neoplasms | 32,238 (22,566 to 45,845) | 2513.1 (1759.1 to 3574) | 1.6 (-0.5 to 4.5) | 17 (14 to 21) | 1.3 (1.1 to 1.6) | -0.8 (-25.4 to 28.4) | 0 (0 to 0) | 0 (0 to 0) | -0.4 (-29 to 39.4) |
|  | Female | Neoplasms | 18,731 (13,228 to 26,802) | 2977.6 (2102.8 to 4260.7) | 1.8 (-0.7 to 5.5) | 1,659 (1,286 to 2,005) | 263.7 (204.4 to 318.7) | -12.4 (-38 to 25.3) | 20 (16 to 24) | 3.2 (2.5 to 3.8) | -12.7 (-37.7 to 24.5) |
|  |  | Other malignant neoplasms | 66 (49 to 88) | 10.5 (7.7 to 13.9) | 170.7 (72.6 to 319.6) | 533 (424 to 663) | 84.7 (67.4 to 105.4) | -2.8 (-33.2 to 42.7) | 6 (5 to 8) | 1 (0.8 to 1.2) | -5.3 (-34.2 to 37.8) |
|  |  | Other neoplasms | 18,601 (13,094 to 26,671) | 2957 (2081.6 to 4239.8) | 1.5 (-1 to 5.2) | 9 (7 to 11) | 1.4 (1.1 to 1.7) | 7 (-24.4 to 44) | 0 (0 to 0) | 0 (0 to 0) | 11.6 (-28.3 to 65) |
|  | Male | Neoplasms | 13,748 (9,455 to 19,393) | 2103 (1446.3 to 2966.6) | 1.4 (-0.2 to 3.5) | 2,042 (1,437 to 2,594) | 312.3 (219.8 to 396.9) | -17.3 (-41.4 to 16.6) | 25 (18 to 32) | 3.9 (2.7 to 4.9) | -17.7 (-41.4 to 15.8) |
|  |  | Other malignant neoplasms | 35 (24 to 47) | 5.3 (3.7 to 7.3) | 31.6 (-12 to 97.2) | 570 (438 to 735) | 87.3 (67 to 112.5) | -12.3 (-39.1 to 25.6) | 7 (5 to 9) | 1.1 (0.8 to 1.4) | -12.5 (-38.7 to 26) |
|  |  | Other neoplasms | 13,636 (9,357 to 19,282) | 2086 (1431.3 to 2949.7) | 1.1 (-0.4 to 3.2) | 8 (6 to 11) | 1.2 (1 to 1.7) | -7.7 (-42.9 to 31.6) | 0 (0 to 0) | 0 (0 to 0) | -10.3 (-48.3 to 41.9) |
| Palestine | Both | Neoplasms | 61,491 (42,776 to 87,491) | 2589.2 (1801.2 to 3684) | 4.1 (1 to 7.1) | 8,927 (7,409 to 11,251) | 375.9 (312 to 473.8) | -34.9 (-52.3 to -5.5) | 111 (92 to 140) | 4.7 (3.9 to 5.9) | -33.7 (-51.3 to -5.3) |
|  |  | Other malignant neoplasms | 91 (74 to 110) | 3.8 (3.1 to 4.6) | 19.7 (-8.5 to 57.5) | 1,804 (1,453 to 2,260) | 76 (61.2 to 95.2) | -23.4 (-43.1 to 4.2) | 23 (18 to 28) | 0.9 (0.8 to 1.2) | -22.3 (-41.7 to 5.3) |
|  |  | Other neoplasms | 61,106 (42,384 to 87,049) | 2573 (1784.7 to 3665.4) | 4.3 (1.2 to 7.5) | 24 (19 to 29) | 1 (0.8 to 1.2) | -0.3 (-21.6 to 24.1) | 0 (0 to 0) | 0 (0 to 0) | -0.6 (-26.2 to 31.6) |
|  | Female | Neoplasms | 35,183 (24,574 to 50,354) | 3040 (2123.3 to 4350.9) | 3.6 (0.4 to 6.6) | 4,011 (3,364 to 4,883) | 346.5 (290.6 to 422) | -32.4 (-54.6 to 5.1) | 49 (42 to 60) | 4.3 (3.6 to 5.2) | -30.9 (-53.5 to 5.5) |
|  |  | Other malignant neoplasms | 54 (40 to 69) | 4.7 (3.5 to 6) | 30.5 (-10.6 to 97.9) | 842 (674 to 1,037) | 72.8 (58.3 to 89.6) | -22.1 (-47.6 to 17.8) | 10 (8 to 13) | 0.9 (0.7 to 1.1) | -21.1 (-46.8 to 17.9) |
|  |  | Other neoplasms | 34,985 (24,395 to 50,166) | 3023 (2107.8 to 4334.6) | 3.8 (0.7 to 6.9) | 12 (10 to 14) | 1 (0.8 to 1.3) | -0.5 (-23.8 to 26.3) | 0 (0 to 0) | 0 (0 to 0) | -1.4 (-29.1 to 33.6) |
|  | Male | Neoplasms | 26,308 (18,119 to 37,311) | 2160.8 (1488.2 to 3064.5) | 4.6 (1.3 to 7.8) | 4,917 (3,778 to 6,804) | 403.8 (310.3 to 558.8) | -36.7 (-59 to 0.9) | 61 (47 to 85) | 5 (3.9 to 7) | -35.7 (-57.7 to 0.7) |
|  |  | Other malignant neoplasms | 37 (28 to 50) | 3 (2.3 to 4.1) | 6.7 (-29.4 to 63.2) | 962 (716 to 1,400) | 79 (58.8 to 115) | -24.5 (-51.9 to 20.8) | 12 (9 to 18) | 1 (0.7 to 1.5) | -23.2 (-50.9 to 22.4) |
|  |  | Other neoplasms | 26,120 (17,893 to 37,095) | 2145.3 (1469.6 to 3046.7) | 4.9 (1.8 to 7.8) | 12 (10 to 15) | 1 (0.8 to 1.2) | -0.1 (-23.1 to 25.2) | 0 (0 to 0) | 0 (0 to 0) | 0.1 (-26.4 to 31.7) |
| Qatar | Both | Neoplasms | 17,196 (11,945 to 24,016) | 3321.4 (2307.1 to 4638.7) | 2.2 (0.6 to 3.7) | 1,340 (1,081 to 1,653) | 258.9 (208.8 to 319.4) | -32.7 (-52.3 to -3.1) | 16 (13 to 20) | 3.2 (2.6 to 3.9) | -33.3 (-52.6 to -4.4) |
|  |  | Other malignant neoplasms | 34 (24 to 45) | 6.5 (4.7 to 8.8) | 102.3 (44.4 to 190.4) | 336 (236 to 439) | 64.9 (45.6 to 84.8) | -18.7 (-40.2 to 13.7) | 4 (3 to 5) | 0.8 (0.5 to 1) | -20.1 (-41.4 to 11.9) |
|  |  | Other neoplasms | 17,101 (11,854 to 23,928) | 3303.2 (2289.6 to 4621.8) | 2.1 (0.4 to 3.6) | 5 (4 to 7) | 1 (0.8 to 1.3) | -7.5 (-27 to 13.4) | 0 (0 to 0) | 0 (0 to 0) | -9.8 (-33.2 to 19) |
|  | Female | Neoplasms | 10,206 (7,105 to 14,490) | 4190.5 (2917.1 to 5949.2) | 1.9 (0.2 to 3.1) | 552 (442 to 694) | 226.8 (181.3 to 284.9) | -29.9 (-50 to 0.4) | 7 (5 to 8) | 2.7 (2.2 to 3.4) | -30.9 (-50.5 to -1.6) |
|  |  | Other malignant neoplasms | 22 (15 to 32) | 9 (6.3 to 13) | 145.6 (57.1 to 278) | 131 (101 to 169) | 53.8 (41.5 to 69.5) | -23.7 (-44.7 to 7.2) | 2 (1 to 2) | 0.6 (0.5 to 0.8) | -27.5 (-47 to 1.4) |
|  |  | Other neoplasms | 10,159 (7,062 to 14,447) | 4171.2 (2899.5 to 5931.6) | 1.7 (0 to 3) | 3 (2 to 3) | 1 (0.8 to 1.3) | -9.4 (-28.8 to 13.8) | 0 (0 to 0) | 0 (0 to 0) | -11.7 (-36.5 to 21.3) |
|  | Male | Neoplasms | 6,989 (4,725 to 9,748) | 2549.2 (1723.6 to 3555.6) | 3.6 (1.3 to 6) | 788 (594 to 1,038) | 287.4 (216.7 to 378.7) | -34.7 (-57.6 to 2.9) | 10 (7 to 13) | 3.6 (2.7 to 4.7) | -35.1 (-57.8 to 1.1) |
|  |  | Other malignant neoplasms | 12 (7 to 17) | 4.3 (2.5 to 6.2) | 52.9 (2.1 to 137.6) | 205 (122 to 291) | 74.8 (44.6 to 106.3) | -15.4 (-46 to 32.8) | 3 (2 to 4) | 0.9 (0.6 to 1.3) | -15.2 (-45.4 to 32) |
|  |  | Other neoplasms | 6,942 (4,671 to 9,701) | 2532.1 (1703.6 to 3538.5) | 3.4 (1.1 to 5.8) | 3 (2 to 4) | 1 (0.8 to 1.3) | -5.6 (-29.5 to 18.9) | 0 (0 to 0) | 0 (0 to 0) | -8 (-37 to 23.6) |
| Saudi Arabia | Both | Neoplasms | 254,583 (176,749 to 355,631) | 2632.2 (1827.5 to 3677) | 4.4 (-0.1 to 9.9) | 17,404 (14,099 to 21,729) | 180 (145.8 to 224.7) | -30.3 (-52 to 4.4) | 218 (177 to 272) | 2.3 (1.8 to 2.8) | -29.3 (-50.6 to 5.2) |
|  |  | Other malignant neoplasms | 350 (268 to 452) | 3.6 (2.8 to 4.7) | 96.4 (38.6 to 177.5) | 4,751 (3,729 to 6,480) | 49.1 (38.6 to 67) | -24 (-44.2 to 5) | 60 (47 to 82) | 0.6 (0.5 to 0.8) | -23.3 (-43.4 to 5) |
|  |  | Other neoplasms | 253,484 (175,924 to 354,599) | 2620.9 (1818.9 to 3666.3) | 4.2 (-0.3 to 9.7) | 73 (56 to 95) | 0.8 (0.6 to 1) | -18.4 (-33.9 to 1.1) | 1 (0 to 1) | 0 (0 to 0) | -25.6 (-44.3 to -1.3) |
|  | Female | Neoplasms | 138,797 (96,331 to 197,973) | 3077.4 (2135.8 to 4389.4) | 3.9 (-0.2 to 9.3) | 8,488 (6,773 to 10,807) | 188.2 (150.2 to 239.6) | -32.7 (-55.8 to 5.5) | 106 (85 to 136) | 2.4 (1.9 to 3) | -31.8 (-54.7 to 7.1) |
|  |  | Other malignant neoplasms | 243 (177 to 332) | 5.4 (3.9 to 7.4) | 133 (45.6 to 263.4) | 2,247 (1,626 to 3,184) | 49.8 (36.1 to 70.6) | -24.6 (-48.2 to 12.3) | 27 (20 to 39) | 0.6 (0.4 to 0.9) | -25.4 (-48.2 to 10.1) |
|  |  | Other neoplasms | 138,209 (95,782 to 197,364) | 3064.3 (2123.7 to 4375.9) | 3.8 (-0.3 to 9.1) | 39 (30 to 51) | 0.9 (0.7 to 1.1) | -14.6 (-34.4 to 9.4) | 0 (0 to 0) | 0 (0 to 0) | -19.3 (-42.5 to 13.7) |
|  | Male | Neoplasms | 115,786 (78,361 to 160,787) | 2243.2 (1518.2 to 3115.1) | 6.3 (1.6 to 12.4) | 8,917 (6,660 to 12,012) | 172.8 (129 to 232.7) | -27.5 (-52.2 to 14.4) | 112 (85 to 151) | 2.2 (1.6 to 2.9) | -26.3 (-50.7 to 15.7) |
|  |  | Other malignant neoplasms | 107 (73 to 161) | 2.1 (1.4 to 3.1) | 47.8 (-16.8 to 126.9) | 2,503 (1,737 to 3,622) | 48.5 (33.6 to 70.2) | -23.4 (-57 to 20.6) | 32 (23 to 46) | 0.6 (0.4 to 0.9) | -21.3 (-55.2 to 23.4) |
|  |  | Other neoplasms | 115,275 (77,819 to 160,337) | 2233.3 (1507.7 to 3106.4) | 6.1 (1.4 to 12) | 34 (25 to 45) | 0.7 (0.5 to 0.9) | -21.7 (-38.9 to -1.7) | 0 (0 to 0) | 0 (0 to 0) | -31.8 (-50.2 to -8) |
| Sudan | Both | Neoplasms | 522,552 (364,304 to 741,894) | 2580.1 (1798.8 to 3663.1) | 3.1 (0.4 to 5.4) | 105,415 (74,476 to 143,987) | 520.5 (367.7 to 710.9) | -35 (-64.2 to 60.1) | 1,291 (921 to 1,751) | 6.4 (4.5 to 8.6) | -32.8 (-62.8 to 61) |
|  |  | Other malignant neoplasms | 727 (504 to 1,019) | 3.6 (2.5 to 5) | 44 (-18.7 to 154.7) | 21,142 (15,220 to 29,131) | 104.4 (75.2 to 143.8) | -15.5 (-52.9 to 55) | 259 (186 to 356) | 1.3 (0.9 to 1.8) | -13.8 (-51.7 to 56.3) |
|  |  | Other neoplasms | 519,241 (361,418 to 737,932) | 2563.8 (1784.5 to 3643.6) | 3.3 (1 to 5.7) | 245 (190 to 323) | 1.2 (0.9 to 1.6) | -2.7 (-50.3 to 61.1) | 3 (2 to 3) | 0 (0 to 0) | -0.7 (-53.2 to 83.8) |
|  | Female | Neoplasms | 297,145 (207,587 to 424,033) | 3029.8 (2116.6 to 4323.5) | 2.2 (-0.3 to 4.2) | 45,141 (31,021 to 65,090) | 460.3 (316.3 to 663.7) | -43.7 (-71.3 to 56.4) | 549 (379 to 796) | 5.6 (3.9 to 8.1) | -41.7 (-69.8 to 58.3) |
|  |  | Other malignant neoplasms | 439 (278 to 653) | 4.5 (2.8 to 6.7) | 64.9 (-21 to 207.9) | 10,075 (6,434 to 14,837) | 102.7 (65.6 to 151.3) | -12.9 (-57.3 to 74.4) | 122 (78 to 179) | 1.2 (0.8 to 1.8) | -11.8 (-56.2 to 75) |
|  |  | Other neoplasms | 295,452 (206,296 to 422,729) | 3012.5 (2103.4 to 4310.2) | 2.6 (0.5 to 4.6) | 114 (86 to 157) | 1.2 (0.9 to 1.6) | -9.1 (-68.5 to 68.8) | 1 (1 to 2) | 0 (0 to 0) | -7.8 (-71.4 to 97.8) |
|  | Male | Neoplasms | 225,407 (155,282 to 318,906) | 2157.9 (1486.6 to 3053) | 4.3 (1.1 to 7.2) | 60,274 (40,356 to 84,515) | 577 (386.4 to 809.1) | -26.5 (-63.6 to 80.7) | 742 (501 to 1,036) | 7.1 (4.8 to 9.9) | -24.2 (-62.2 to 82.4) |
|  |  | Other malignant neoplasms | 288 (198 to 425) | 2.8 (1.9 to 4.1) | 20.7 (-45.9 to 152.1) | 11,067 (7,693 to 16,268) | 105.9 (73.6 to 155.7) | -17.7 (-62.4 to 71.1) | 137 (95 to 199) | 1.3 (0.9 to 1.9) | -15.5 (-61.2 to 74.5) |
|  |  | Other neoplasms | 223,789 (153,550 to 317,105) | 2142.4 (1470 to 3035.8) | 4.4 (1.7 to 7.1) | 131 (100 to 177) | 1.3 (1 to 1.7) | 3.7 (-47.7 to 82.6) | 1 (1 to 2) | 0 (0 to 0) | 6 (-50.2 to 108.8) |
| Syrian Arab republic | Both | Neoplasms | 161,646 (112,065 to 226,740) | 2746.7 (1904.2 to 3852.7) | 8.5 (0.6 to 18.5) | 26,198 (20,127 to 38,885) | 445.2 (342 to 660.7) | -34 (-54.1 to -4.4) | 331 (254 to 490) | 5.6 (4.3 to 8.3) | -31.9 (-52.3 to -1.9) |
|  |  | Other malignant neoplasms | 224 (172 to 292) | 3.8 (2.9 to 5) | 82.8 (33.1 to 157.3) | 3,625 (2,762 to 4,619) | 61.6 (46.9 to 78.5) | -4.1 (-32.2 to 34.5) | 45 (34 to 58) | 0.8 (0.6 to 1) | -2.5 (-30.8 to 36.2) |
|  |  | Other neoplasms | 160,540 (110,980 to 225,689) | 2727.9 (1885.8 to 3834.9) | 8.7 (0.8 to 18.8) | 77 (63 to 95) | 1.3 (1.1 to 1.6) | 11.5 (-21.3 to 46.9) | 1 (1 to 1) | 0 (0 to 0) | 17.9 (-21.6 to 63) |
|  | Female | Neoplasms | 92,318 (63,540 to 131,867) | 3191.8 (2196.8 to 4559.2) | 7.1 (-0.8 to 17.4) | 10,943 (8,490 to 17,322) | 378.3 (293.5 to 598.9) | -35.6 (-55.6 to 3.4) | 138 (107 to 216) | 4.8 (3.7 to 7.5) | -33.3 (-54 to 5.4) |
|  |  | Other malignant neoplasms | 145 (105 to 191) | 5 (3.6 to 6.6) | 117.4 (52.3 to 216.3) | 1,705 (1,295 to 2,149) | 58.9 (44.8 to 74.3) | -0.2 (-27.5 to 37) | 21 (16 to 27) | 0.7 (0.6 to 0.9) | 0.5 (-26.4 to 37.5) |
|  |  | Other neoplasms | 91,791 (63,010 to 131,340) | 3173.6 (2178.5 to 4540.9) | 7.3 (-0.6 to 17.7) | 43 (33 to 55) | 1.5 (1.1 to 1.9) | 13.2 (-31.7 to 64.2) | 0 (0 to 1) | 0 (0 to 0) | 19.6 (-35.1 to 86.8) |
|  | Male | Neoplasms | 69,328 (46,290 to 95,689) | 2316.5 (1546.7 to 3197.3) | 10.1 (2.1 to 20.1) | 15,255 (10,958 to 23,600) | 509.7 (366.1 to 788.5) | -32.7 (-56.1 to 14) | 193 (139 to 298) | 6.5 (4.6 to 10) | -30.8 (-54.6 to 16.1) |
|  |  | Other malignant neoplasms | 79 (55 to 108) | 2.7 (1.8 to 3.6) | 41.5 (-5.7 to 113.4) | 1,920 (1,321 to 2,514) | 64.2 (44.1 to 84) | -7.2 (-42 to 44.3) | 24 (17 to 32) | 0.8 (0.6 to 1.1) | -4.9 (-40 to 47.3) |
|  |  | Other neoplasms | 68,749 (45,659 to 95,218) | 2297.1 (1525.6 to 3181.6) | 10.3 (2.4 to 20.3) | 35 (28 to 42) | 1.2 (0.9 to 1.4) | 9.3 (-18.3 to 39.2) | 0 (0 to 0) | 0 (0 to 0) | 15.8 (-17.7 to 53.7) |
| Tunisia | Both | Neoplasms | 89,864 (62,626 to 127,507) | 2603.4 (1814.3 to 3693.9) | 1.3 (0.1 to 2.6) | 7,088 (5,588 to 8,887) | 205.3 (161.9 to 257.4) | -44.3 (-60 to -19.5) | 87 (69 to 110) | 2.5 (2 to 3.2) | -43.6 (-59.4 to -19.4) |
|  |  | Other malignant neoplasms | 147 (105 to 198) | 4.3 (3 to 5.7) | 43.5 (-2.4 to 113) | 2,071 (1,509 to 2,710) | 60 (43.7 to 78.5) | -32.5 (-53.1 to -5) | 25 (18 to 34) | 0.7 (0.5 to 1) | -32.2 (-52.8 to -5) |
|  |  | Other neoplasms | 89,455 (62,183 to 127,056) | 2591.5 (1801.4 to 3680.8) | 1.4 (0.2 to 2.6) | 40 (31 to 51) | 1.2 (0.9 to 1.5) | -18.6 (-42.2 to 5.2) | 0 (0 to 0) | 0 (0 to 0) | -23.3 (-49.7 to 5.8) |
|  | Female | Neoplasms | 50,875 (35,508 to 72,749) | 3059.6 (2135.4 to 4375.1) | 1.3 (0.1 to 2.6) | 3,554 (2,740 to 4,533) | 213.7 (164.8 to 272.6) | -49.9 (-65.8 to -26.2) | 44 (34 to 56) | 2.6 (2 to 3.4) | -49.1 (-64.8 to -25.3) |
|  |  | Other malignant neoplasms | 104 (70 to 147) | 6.3 (4.2 to 8.9) | 60.5 (-4.2 to 156.5) | 1,105 (799 to 1,483) | 66.4 (48 to 89.2) | -33.7 (-57.1 to 2) | 13 (10 to 18) | 0.8 (0.6 to 1.1) | -34 (-57.2 to 1.1) |
|  |  | Other neoplasms | 50,640 (35,290 to 72,521) | 3045.5 (2122.3 to 4361.4) | 1.3 (0.1 to 2.6) | 19 (15 to 25) | 1.2 (0.9 to 1.5) | -22.1 (-44.9 to 7.3) | 0 (0 to 0) | 0 (0 to 0) | -27.6 (-53.3 to 7.7) |
|  | Male | Neoplasms | 38,988 (26,641 to 55,287) | 2179.3 (1489.1 to 3090.3) | 1.9 (0.7 to 3.1) | 3,533 (2,679 to 4,607) | 197.5 (149.7 to 257.5) | -36.9 (-58.1 to -1.2) | 44 (33 to 57) | 2.4 (1.8 to 3.2) | -36.4 (-57.8 to -1.3) |
|  |  | Other malignant neoplasms | 43 (28 to 64) | 2.4 (1.6 to 3.6) | 15.2 (-28.6 to 97.3) | 966 (655 to 1,404) | 54 (36.6 to 78.5) | -30.7 (-58.1 to 12.5) | 12 (8 to 18) | 0.7 (0.5 to 1) | -29.9 (-57.5 to 12.4) |
|  |  | Other neoplasms | 38,814 (26,446 to 55,096) | 2169.6 (1478.2 to 3079.6) | 1.9 (0.7 to 3) | 21 (16 to 27) | 1.2 (0.9 to 1.5) | -15 (-43.9 to 16.3) | 0 (0 to 0) | 0 (0 to 0) | -19.1 (-51.5 to 18.6) |
| Turkey | Both | Neoplasms | 690,801 (469,575 to 953,816) | 3098.5 (2106.2 to 4278.2) | 3.6 (0 to 7.9) | 96,940 (78,913 to 115,662) | 434.8 (354 to 518.8) | -51.3 (-64.5 to -28.7) | 1,193 (972 to 1,420) | 5.4 (4.4 to 6.4) | -51 (-63.7 to -29.6) |
|  |  | Other malignant neoplasms | 1,346 (1,102 to 1,647) | 6 (4.9 to 7.4) | 46.6 (7.4 to 98.7) | 21,482 (17,872 to 25,822) | 96.4 (80.2 to 115.8) | -35.7 (-53 to -12.2) | 265 (221 to 316) | 1.2 (1 to 1.4) | -35.4 (-52.6 to -12.5) |
|  |  | Other neoplasms | 685,229 (463,409 to 948,455) | 3073.5 (2078.6 to 4254.2) | 3.7 (0.1 to 8) | 1,089 (732 to 1,456) | 4.9 (3.3 to 6.5) | -35.3 (-60.1 to -3) | 11 (7 to 16) | 0.1 (0 to 0.1) | -38 (-62.5 to -2.5) |
|  | Female | Neoplasms | 388,923 (264,704 to 539,849) | 3585.8 (2440.5 to 4977.3) | 2.9 (-0.6 to 7.1) | 42,957 (34,084 to 51,376) | 396.1 (314.2 to 473.7) | -54 (-69.6 to -29.1) | 529 (422 to 630) | 4.9 (3.9 to 5.8) | -53.2 (-68.5 to -29.2) |
|  |  | Other malignant neoplasms | 851 (666 to 1,071) | 7.8 (6.1 to 9.9) | 49 (-1.8 to 118.4) | 9,895 (7,815 to 12,157) | 91.2 (72 to 112.1) | -46.1 (-65.4 to -20.7) | 121 (96 to 149) | 1.1 (0.9 to 1.4) | -46.3 (-65 to -21.2) |
|  |  | Other neoplasms | 386,441 (262,289 to 537,154) | 3562.9 (2418.2 to 4952.4) | 3 (-0.4 to 7.3) | 580 (403 to 774) | 5.3 (3.7 to 7.1) | -30.7 (-67.9 to 27) | 6 (4 to 8) | 0.1 (0 to 0.1) | -33.4 (-70.9 to 28.7) |
|  | Male | Neoplasms | 301,879 (204,589 to 417,365) | 2636.9 (1787.1 to 3645.6) | 4.7 (0.7 to 9.1) | 53,983 (43,429 to 65,538) | 471.5 (379.3 to 572.5) | -49 (-66.7 to -22.1) | 664 (537 to 809) | 5.8 (4.7 to 7.1) | -49 (-66.3 to -22.3) |
|  |  | Other malignant neoplasms | 495 (386 to 629) | 4.3 (3.4 to 5.5) | 43 (0.6 to 110) | 11,587 (9,138 to 14,676) | 101.2 (79.8 to 128.2) | -23 (-46.1 to 12.2) | 144 (114 to 183) | 1.3 (1 to 1.6) | -22.2 (-44.7 to 12.7) |
|  |  | Other neoplasms | 298,789 (201,268 to 414,227) | 2609.9 (1758.1 to 3618.2) | 4.7 (0.7 to 9) | 509 (231 to 799) | 4.4 (2 to 7) | -39.9 (-65.3 to 8) | 5 (2 to 9) | 0 (0 to 0.1) | -42.5 (-66.4 to 4.4) |
| United Arab Emirates | Both | Neoplasms | 39,853 (27,553 to 57,148) | 2629.4 (1817.9 to 3770.6) | 6.5 (1.5 to 12.2) | 4,487 (3,404 to 5,613) | 296 (224.6 to 370.3) | -39.4 (-56.7 to -17.4) | 57 (43 to 70) | 3.7 (2.8 to 4.6) | -37.9 (-55.3 to -15.7) |
|  |  | Other malignant neoplasms | 66 (48 to 91) | 4.4 (3.1 to 6) | 10.8 (-19.2 to 52.1) | 1,249 (943 to 1,570) | 82.4 (62.2 to 103.6) | -35.9 (-55.7 to -11.6) | 16 (12 to 20) | 1 (0.8 to 1.3) | -34.6 (-54.4 to -9.7) |
|  |  | Other neoplasms | 39,660 (27,371 to 56,898) | 2616.7 (1805.9 to 3754.1) | 6.5 (1.5 to 12.3) | 19 (15 to 24) | 1.3 (1 to 1.6) | -38.2 (-59.8 to -8.9) | 0 (0 to 0) | 0 (0 to 0) | -43.8 (-66.2 to -8.9) |
|  | Female | Neoplasms | 22,671 (15,731 to 32,723) | 3082.9 (2139.2 to 4449.9) | 5.7 (0.4 to 11.8) | 1,933 (1,410 to 2,576) | 262.9 (191.7 to 350.3) | -33.3 (-54.2 to -2.4) | 24 (18 to 32) | 3.3 (2.4 to 4.4) | -31.8 (-53 to -1.3) |
|  |  | Other malignant neoplasms | 43 (26 to 69) | 5.9 (3.6 to 9.4) | 31.8 (-16.4 to 109.4) | 584 (369 to 876) | 79.4 (50.2 to 119.2) | -32.1 (-53.2 to -0.7) | 7 (5 to 11) | 1 (0.6 to 1.4) | -31.1 (-51.9 to 0) |
|  |  | Other neoplasms | 22,571 (15,646 to 32,603) | 3069.3 (2127.7 to 4433.6) | 5.7 (0.4 to 11.9) | 9 (7 to 12) | 1.2 (1 to 1.6) | -23.8 (-48.5 to 6.6) | 0 (0 to 0) | 0 (0 to 0) | -28.5 (-54 to 11.7) |
|  | Male | Neoplasms | 17,182 (11,666 to 24,561) | 2202.1 (1495.1 to 3147.7) | 7 (2.3 to 12.4) | 2,553 (1,769 to 3,501) | 327.2 (226.8 to 448.8) | -43.1 (-61.4 to -15.8) | 32 (23 to 44) | 4.1 (2.9 to 5.7) | -41.6 (-60 to -14.5) |
|  |  | Other malignant neoplasms | 23 (15 to 32) | 3 (1.9 to 4.1) | -14.8 (-55.3 to 38) | 665 (420 to 948) | 85.3 (53.8 to 121.4) | -38.9 (-70 to 3.6) | 8 (5 to 12) | 1.1 (0.7 to 1.5) | -37.1 (-69 to 5) |
|  |  | Other neoplasms | 17,089 (11,567 to 24,425) | 2190.2 (1482.4 to 3130.4) | 7.2 (2.5 to 12.5) | 10 (8 to 13) | 1.3 (1 to 1.6) | -47.1 (-72.1 to -6.1) | 0 (0 to 0) | 0 (0 to 0) | -52.3 (-76.5 to -5) |
| Yemen | Both | Neoplasms | 418,792 (291,490 to 595,847) | 2567 (1786.7 to 3652.2) | 4.5 (0.8 to 9.1) | 64,196 (44,096 to 88,373) | 393.5 (270.3 to 541.7) | -20.5 (-53.5 to 76.2) | 787 (543 to 1,082) | 4.8 (3.3 to 6.6) | -17.8 (-51.9 to 80.7) |
|  |  | Other malignant neoplasms | 438 (312 to 599) | 2.7 (1.9 to 3.7) | 48.5 (-7 to 149.5) | 13,744 (9,682 to 18,744) | 84.2 (59.3 to 114.9) | 2.9 (-39.1 to 76) | 168 (118 to 229) | 1 (0.7 to 1.4) | 5 (-37.3 to 78.7) |
|  |  | Other neoplasms | 416,893 (289,481 to 594,173) | 2555.3 (1774.4 to 3642) | 4.6 (1 to 9.1) | 159 (118 to 204) | 1 (0.7 to 1.2) | 10.3 (-29.2 to 63.2) | 2 (1 to 2) | 0 (0 to 0) | 16.7 (-32.5 to 92.7) |
|  | Female | Neoplasms | 239,976 (168,081 to 344,561) | 3017.4 (2113.4 to 4332.4) | 3.7 (0 to 8.3) | 30,629 (19,941 to 43,861) | 385.1 (250.7 to 551.5) | -25.6 (-58.1 to 80.6) | 372 (242 to 532) | 4.7 (3 to 6.7) | -23 (-57.2 to 82.1) |
|  |  | Other malignant neoplasms | 271 (186 to 393) | 3.4 (2.3 to 4.9) | 68.8 (-5.2 to 189.3) | 7,061 (4,876 to 9,868) | 88.8 (61.3 to 124.1) | 7.6 (-40.6 to 90.5) | 86 (59 to 120) | 1.1 (0.7 to 1.5) | 9.4 (-39.1 to 94.4) |
|  |  | Other neoplasms | 238,936 (167,173 to 343,409) | 3004.3 (2102 to 4317.9) | 3.8 (0.1 to 8.5) | 79 (58 to 104) | 1 (0.7 to 1.3) | 8.7 (-51 to 69.6) | 1 (1 to 1) | 0 (0 to 0) | 15.9 (-54.7 to 109.9) |
|  | Male | Neoplasms | 178,816 (122,676 to 253,817) | 2138.5 (1467.1 to 3035.5) | 4.8 (1.2 to 9.4) | 33,567 (21,517 to 47,256) | 401.4 (257.3 to 565.2) | -15.2 (-55.3 to 93.9) | 415 (267 to 582) | 5 (3.2 to 7) | -12.6 (-53.8 to 98.7) |
|  |  | Other malignant neoplasms | 167 (111 to 247) | 2 (1.3 to 3) | 23.9 (-40 to 140.8) | 6,683 (4,483 to 9,945) | 79.9 (53.6 to 118.9) | -1.7 (-52.3 to 91.7) | 83 (56 to 121) | 1 (0.7 to 1.4) | 0.8 (-50.8 to 96.5) |
|  |  | Other neoplasms | 177,957 (121,827 to 252,869) | 2128.3 (1457 to 3024.2) | 4.9 (1.3 to 9.4) | 80 (59 to 108) | 1 (0.7 to 1.3) | 11.8 (-34.7 to 82.8) | 1 (1 to 1) | 0 (0 to 0) | 17.6 (-36.7 to 120.2) |

*Data in parentheses are 95% Uncertainty Intervals (95% UIs); DALYs= Disability-Adjusted Life Years*
